# Quantum-Chemical Consideration of Al_2_M_2_ Tetranuclear Metal Clusters (M–3*d*-Element): Molecular/Electronic Structures and Thermodynamics

**DOI:** 10.3390/ma14226836

**Published:** 2021-11-12

**Authors:** Oleg V. Mikhailov, Denis V. Chachkov

**Affiliations:** 1 Department of Analytical Chemistry, Certification and Quality Management, Kazan National Research Technological University, K. Marx Street 68, 420015 Kazan, Russia; 2 Kazan Department of Joint Supercomputer Center of Russian Academy of Sciences—Branch of Federal Scientific Center “Scientific Research Institute for System Analysis of the RAS”, Lobachevskii Street 2/31, 420111 Kazan, Russia; de2005c@gmail.com

**Keywords:** metal cluster, aluminum, scandium, titanium, vanadium, chromium, manganese, iron, cobalt, nickel, copper, zinc, molecular structure, thermodynamic parameters, DFT method

## Abstract

Quantum-chemical calculation of most important parameters of molecular and electronic structures of tetra-nuclear (*pd*) metal clusters having Al_2_M_2_ composition, where M = Sc, Ti, V, Cr, Mn, Fe, Co, Ni, Cu, or Zn (bond lengths, bond and torsion angles), and HOMO and LUMO of these compounds by means of DFT OPBE/QZVP method, have been carried out. It has been found that, for each of these metal clusters, an existence of rather large amount of structural isomers different substantially in their total energy, occurs. It has been noticed that molecular structures of metal clusters of the given type differ significantly between them in terms of geometric parameters, as well as in geometric form, wherein the most stable modifications of metal clusters considered are similar between themselves in geometric form. In addition, the standard thermodynamic parameters of formation of metal clusters considered here, and namely standard enthalpy Δ_f_*H*^0^(298 K), entropy S_f_^0^(298 K), and Gibbs’ energy Δ_f_*G*^0^(298 K) of formation for these metal clusters, were calculated.

## 1. Introduction

Hetero-element metal clusters containing atoms of various *p*- and *d*-elements, at the present time already found a number applications in the various fields of science and technique (see, in particular, References [1,2,3,4,5]). Currently, there are a number of theoretical research studies of the given interesting objects; as a result of such research studies by means of quantum-chemical calculations (as a rule, using the density functional theory (DFT)), the data of their molecular structures and other physicochemical characteristics were found [6,7,8,9,10,11,12,13,14,15,16,17,18,19,20,21,22,23,24]. In the given publications, however, bi-element (*dd*)metal clusters containing various sets of 3*d*-, 4*d*-, and 5*d*-metals included in one period (in particular, Cu_n_Fe [6], Ag_n_Pd_m_ [7,8,9], Au_n_Ir [3]), as well as in different periods of the D.I. Mendeleev’s Periodic Table of Chemical Elements (for example, FePd_n_ [10], Pt_n_Cu_m_ [11], Au_n_Fe [12], Au_n_Pd_m_ [13], Au_n_Ag_m_ [14]) (n and m are different integers)), were considered. At the same time, (*pd*)hetero-element metal clusters containing atoms of different *p*- and *d*-elements were studied much less frequently [15,16,17,18,19,20,21,22,23,24], despite the fact that, a priori, it may be expected that they would have certain novel properties which would be absent in metal clusters containing only d-element atoms. As is known, the most widespread and widely used of all p-metallic elements is Al, and it is no coincidence that in all publications devoted to (pd)metal clusters, namely, this p-element was included in the composition of each of such metal clusters. In the works [15,16,17,18,19,20,21,22,23,24] cited above, however, the objects of research were penta-nuclear or hexa-nuclear (AlM) metal clusters. The review article by Reference [25] was devoted to systematization and generalization of the results of these studies. Tetra-nuclear metal clusters of the Al_2_M_2_ type, where M is any *d*-element, as far as we know, till now, were considered only in the work [26]. The point is that, on the one hand, owing to the small number of atoms in their molecules (only 4), for their molecular structures, one can, a priori, expect only a very small variety of geometric forms; the circumstance indicated makes such objects of little interest for structural chemistry. However, a closer examination of the molecular structures of (*pd*)metal clusters of the Al_2_M_2_ type, for various 3*d*-elements, allows us to assert the existence of a very significant number of structural isomers and a much larger assortment of molecular structures than it might seem at first sight. On the other hand, in all the works [15,16,17,18,19,20,21,22,23,24,25,26] indicated above, quantum-chemical calculations were carried out either the DFT method with the OPBE/TZVP level, or methods of lower-level. Taking into account these two important circumstances, this article will be devoted to the presentation and systematization of the results of quantum-chemical calculations of the molecular structures of Al_2_M_2_ metal clusters (where M is each of ten 3*d*-elements) obtained by a more perfect version of DFT method compared to those used in References [15,16,17,18,19,20,21,22,23,24,25,26] and, namely, DFT OPBE/QZVP.

## 2. Results

According to our data obtained by DFT OPBE/QZVP method, each of the tetranuclear Al_2_M_2_ metal clusters forms a number of structural isomers, the amount of which varies from 6 (Al_2_Ni_2_) to 22 (Al_2_Fe_2_) (Table 1). In this connection, we note that, according to the data obtained as a result of calculations using the DFT OPBE/TZVP method, for the Al_2_Fe_2_ metal cluster, a much smaller number of structural isomers is realized, namely 12 [25,26].

Information on the relative energies of all Al_2_M_2_ type metal clusters (M = Sc, Ti, V, Cr, Mn, Fe, Co, Ni, Cu, or Zn) identified as a result of our calculation is presented in Tables S1–S10 of the Supplementary Materials (in terms of the numbering of metal clusters, the Arabic numeral in parentheses denotes the value of the spin multiplicity of the ground state (*M_S_*), and the Roman numeral is the ordinal number of the metal cluster with *M_S_* data in ascending relative energy. Structural isomers marked with (*) are biradical, i.e., have two unpaired electrons with a total spin S = 0 (and, hence, *M_S_* = 1). The key parameters of the molecular structures of metal clusters under study with the lowest total energy (i.e., the most energetically stable), namely interatomic distances, planar, and torsion angles between different atoms, are presented in Table 2 and Table 3.

**Figure 1:**
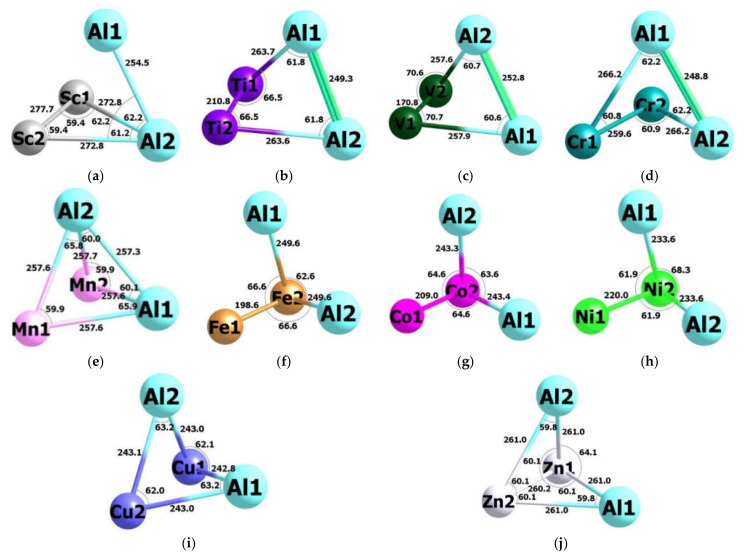
The images of most stable Al_2_M_2_ metal clusters: (**a**)—Al_2_Sc_2_ (3-I), (**b**)—Al_2_Ti_2_ (3-I), (**c**)—Al_2_V_2_ (3-I), (**d**)—Al_2_Cr_2_ (1-I)*, (**e**)—Al_2_Mn_2_ (1-I)*, (**f**)—Al_2_Fe_2_ (5-I), (**g**)—Al_2_Co_2_ (5-I), (**h**)—Al_2_Ni_2_ (1-I), (**i**)—Al_2_Cu_2_ (1-I), (**j**)—Al_2_Zn_2_ (1-I)*.

Stylized images of the molecular structures of each of these most stable metal clusters are shown in Figure 1; a complete assortment of molecular structures of all Al_2_M_2_ metal clusters under consideration can be found in the Supplementary Materials. The values of the standard thermodynamic parameters for the most stable from each of Al_2_M_2_ metal clusters (standard enthalphy Δ_f_*H*^0^(298 K), standard entropy S_f_^0^(298 K), and standard Gibbs’s energy Δ_f_*G*^0^(298 K) of formation) calculated within the DFT OPBE/QZVP method are given in Table 4. NBO analysis data for each of such metal clusters are presented in Table 5.

**Figure 2:**
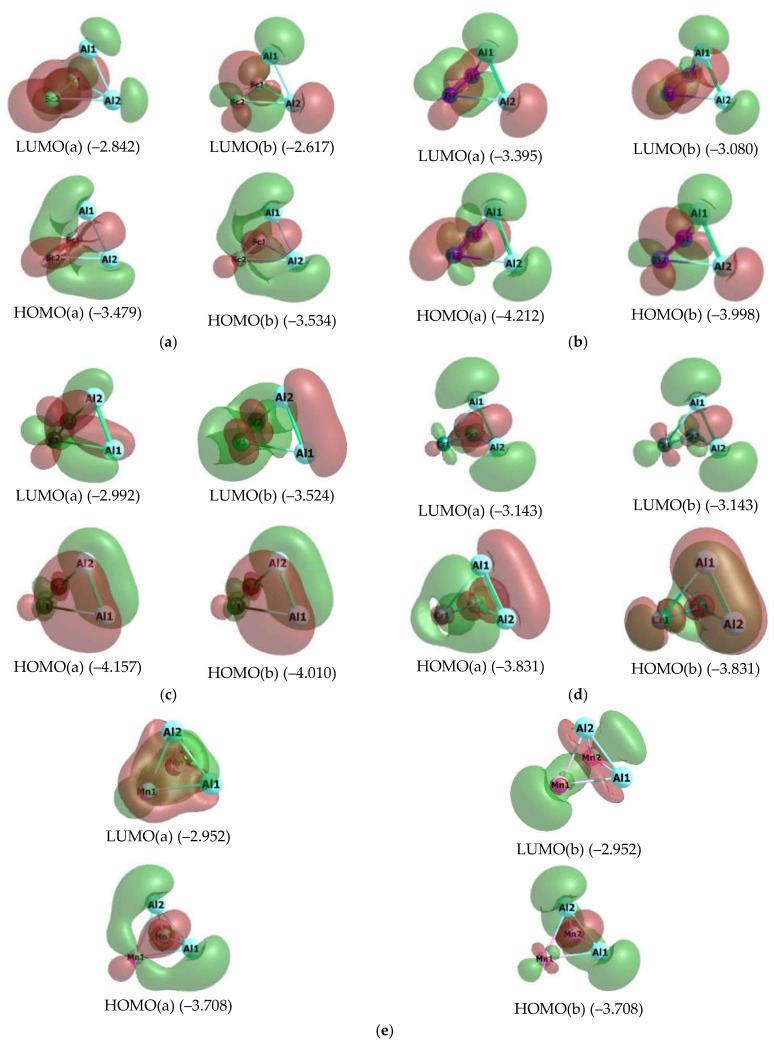
Images of highest occupied (HOMO) and lowest unoccupied (LUMO) molecular orbitals in the Al_2_Sc_2_ (3-I) (**a**), Al_2_Ti_2_ (3-I) (**b**), Al_2_V_2_ (3-I) (**c**), Al_2_Cr_2_ (1-I)* (**d**), and Al_2_Mn_2_ (1-I)* (**e**) metal clusters. The values of energies of these molecular orbitals (in brackets) are given in eV. The symbol “a” corresponds to electron with spin (+1/2), “b”, to electron with spin–l/2).

**Figure 3:**
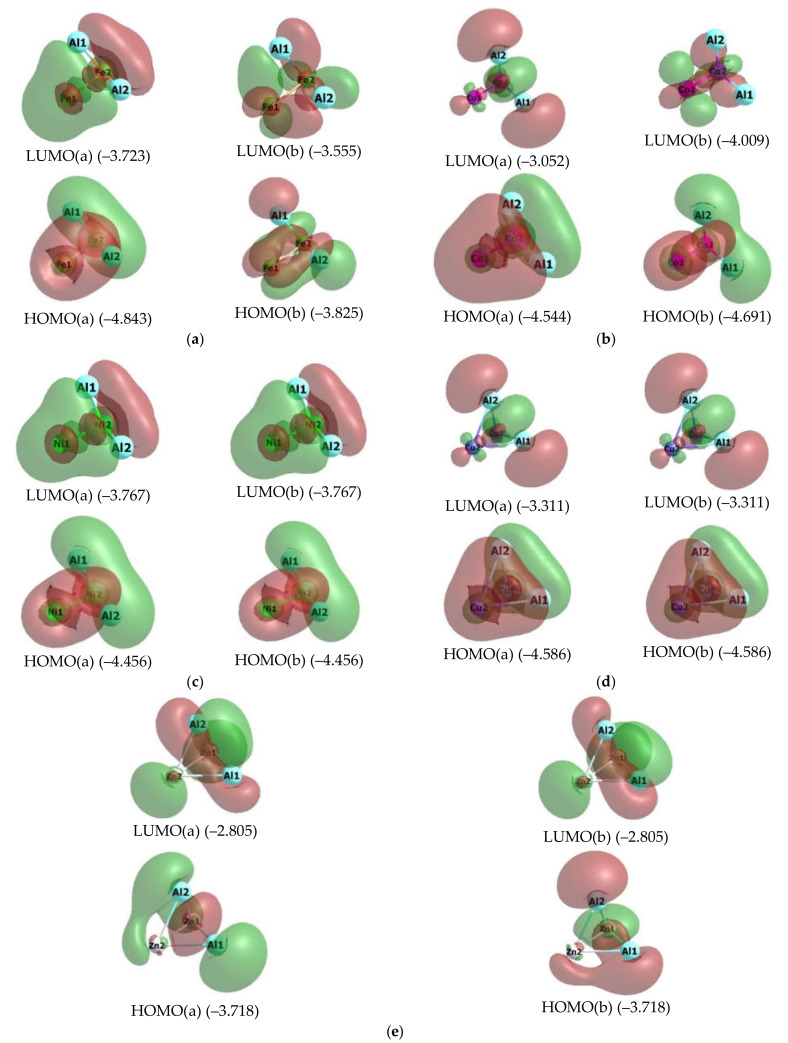
Images of highest occupied (HOMO) and lowest unoccupied (LUMO) molecular orbitals in the Al_2_Fe_2_ (5-I) (**a**), Al_2_Co_2_ (5-I) (**b**), Al_2_Ni_2_ (1-I) (**c**), Al_2_Cu_2_ (1-I) (**d**), and Al_2_Zn_2_ (1-I)* (**e**) metal clusters. The values of energies of these molecular orbitals (in brackets) are given in eV. The symbol “a” corresponds to electron with spin (+1/2), “b”, to electron with spin (–l/2).

**Figure 4:**
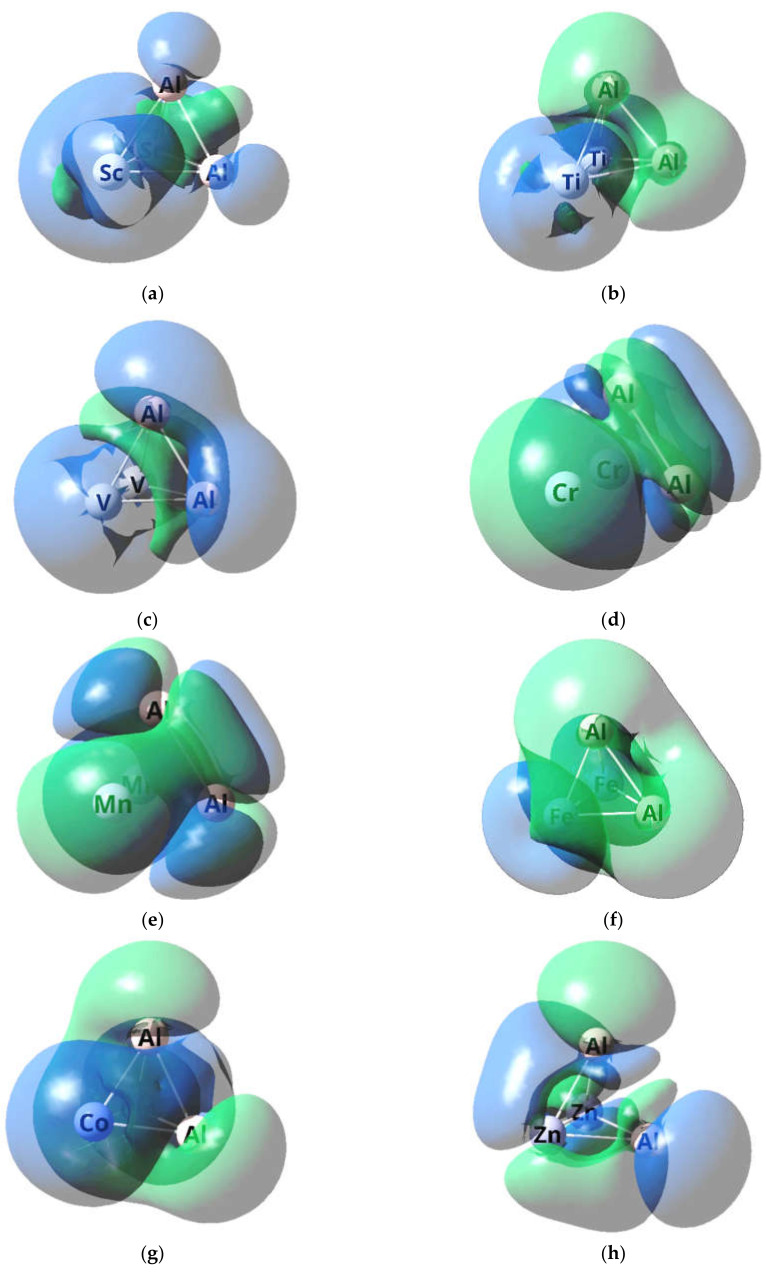
The pattern of distribution of the spin density in the Al_2_Sc_2_ (3-I) (**a**), Al_2_Ti_2_ (3-I) (**b**), Al_2_V_2_ (3-I) (**c**), Al_2_Cr_2_ (1-I)* (**d**), Al_2_Mn_2_ (1-I)* (**e**), Al_2_Fe_2_ (5-I) (**f**), Al_2_Co_2_ (5-I) (**g**), and Al_2_Zn_2_ (1-I)* (**h**) metal clusters. For Al_2_Ni_2_ (1-I) and Al_2_Cu_2_ (1-I), these pictures are absent since, for them, <S**2> = 0.

The images of higher occupied and low unoccupied (vacant) molecular orbitals (HOMO and LUMO, respectively) for the metal clusters under consideration are presented in Figure 2 and Figure 3, while the pattern of distribution of the spin density in these metal clusters is given in Figure 4.

## 3. Discussion 

As it can be seen from Table 1, the number of possible structural isomers of metal clusters of the Al_2_M_2_ type depends very significantly on the nature of the 3*d*-element M, which is in the composition of this metal cluster; wherein, that is characteristic, no clear-cut regularity is observed between the specificity of the electronic configuration of the 3*d*-element and the number of structural isomers. To a first approximation, all these structural isomers can be subdivided into two categories—planar (two-dimensional) and “voluminous” (three-dimensional). That is characteristic, and ongoing from Al_2_Sc_2_ to Al_2_Zn_2_, there is a gradual increase in the proportion of structural isomers with a planar or close to it space structure in the total mass of structural isomers. So, if, in the case of Al_2_Sc_2_, among its 7 isomers, there is none with a planar structure, then, in the case of Al_2_Ti_2_, there are 2 such structures of 13 (Al_2_Ti_2_ (1-VII) and Al_2_Ti_2_ (1-VIII)), in the case of Al_2_V_2_, 3 of 14 (Al_2_V_2_ (1-VII), Al_2_V_2_ (3-II), Al_2_V_2_ (5-III)), in the case of Al_2_Fe_2_—already 11 out of 22 (Al_2_Fe_2_ (1-III), Al_2_Fe_2_ (1-VII), Al_2_Fe_2_ (1-VIII), Al_2_Fe_2_ (1-X), Al_2_Fe_2_ (3-III)—Al_2_Fe_2_ (3-VI), Al_2_Fe_2_ (5-IV)—Al_2_Fe_2_ (5-VI)), i.e., exactly half of their total number; a similar situation occurs for cobalt, nickel, copper, and zinc metal clusters (see Supplementary Materials). Nevertheless, all of the lowest-energy Al_2_M_2_ clusters have a three-dimensional structure that, to one degree or another, resembles a tetrahedron or trigonal pyramid (Figure 1). Theoretically, of course, none of the metal clusters under consideration should have the configuration of a regular tetrahedron due to the nonequivalence of the Al atoms and the atoms of the 3*d*-element M; the closest to this are the Al_2_Mn_2_ (1-I) and Al_2_Zn_2_ (1-I) structures, each of which has five metal-metal chemical bonds with four Al–M bonds of the same length and one Al–Al bond (in the case of Al_2_Mn_2_ (1-I)) or M–M bond (in the case of Al_2_Zn_2_ (1-I)) (Figure 1). It is very remarkable that such structural isomers are observed for metal clusters of, namely, such 3*d*-elements since those atoms have either a half (Mn) or a fully filled (Zn) with electrons 3*d*-sublevel (3*d*^5^ and 3*d*^10^, respectively) with a uniform distribution of electron density in space. The indicated number of metal–metal chemical bonds is the maximum among the Al_2_M_2_ metal clusters, in general, and the most energetically favourable, in particular; for the latter, the number of metal–metal bonds is either 4 (in Al_2_Sc_2_ (3-I), Al_2_Ti_2_ (3-I), Al_2_V_2_ (3-I), Al_2_Cr_2_ (1-I), and Al_2_Cu_2_ (1-I)), or 3 (in Al_2_Fe_2_ (5-I), Al_2_Co_2_ (5-I), and Al_2_Ni_2_ (1-I)) (Figure 1). It is interesting that, in the case of Al_2_Fe_2_, there are two structural isomers with five metal–metal bonds and with higher total energies than the lowest-energy Al_2_Fe_2_ (5-I) metal cluster, namely Al_2_Fe_2_ (1-I) and Al_2_Fe_2_ (1-II), in each of which, as in Al_2_Zn_2_ (1-I), Al–Al bond is absent (see Supplementary Materials).

Planar structural isomers of Al_2_M_2_ exhibit a much greater variety in geometric shapes than three-dimensional ones. Among them, there are isomers in the form of a rhombus, a trapezoid, an irregular quadrangle, a triangular star, a trident (at the vertices of which there can be both Al atoms and M atoms), and, also, a zigzag (see Supplementary Materials, Figures S1–S10). Structural isomers having the rhombic form are very rare and are observed only among Al_2_Cu_2_ metal clusters (Al_2_Cu_2_ (5-I) and Al_2_Cu_2_ (5-IV) with metal—metal bond lengths 238.3 and 240.4 pm, respectively) and among Al_2_Zn_2_ metal clusters (Al_2_Zn_2_ (1-IV) and Al_2_Zn_2_ (3-II) with metal–metal bond lengths of 259.1 and 259.7 pm, respectively). The trapezoid shape is noted only for the metal clusters Al_2_Ti_2_ (1-VIII) and Al_2_Zn_2_ (5-I); similar forms are observed for metal clusters Al_2_Ti_2_ (1-VII), Al_2_V_2_ (3-II), Al_2_Cr_2_ (3-III), Al_2_Mn_2_ (5-VI), Al_2_Fe_2_ (5-IV), Al_2_Fe_2_ (5-V), and Al_2_Co_2_ (1-IX). The shape of a triangular star is represented in structural isomers Al_2_Mn_2_ (5-V), Al_2_Mn_2_ (7-II), Al_2_Fe_2_ (3-V), Al_2_Fe_2_ (5-VI), and Al_2_Cu_2_ (5-V); in this case, that is characteristic, and, in the first four of these five metal clusters, the central atom is the M atom (Mn and Fe, respectively), while, in the latter, the Al atom (see Figures S5, S6, and S9). The planar trident shape is especially characteristic of the Al_2_Cr_2_ metal clusters, where it is represented by three structural isomers Al_2_Cr_2_ (1-III), Al_2_Cr_2_ (3-II), and Al_2_Cr_2_ (5-II); however, in vertices of the first and third isomers, Cr atoms are, and the second isomer, it is Al atoms. Note in this connection that, in Al_2_Cr_2_ (3-II), the Cr atoms are in the *cis*-position relative to each other; it is noteworthy that a trident structure with M atoms in *trans*-positions was not found either for the Al_2_Cr_2_ metal cluster, or any other of the metal clusters under study. Finally, the zigzag shape takes place for the metal clusters Al_2_Mn_2_ (5-VII), Al_2_Fe_2_ (1-III), Al_2_Fe_2_ (3-IV), Al_2_Co_2_ (1-V), Al_2_Co_2_ (1-VII), Al_2_Ni_2_ (5-II), Al_2_Cu_2_ (3-III), Al_2_Cu_2_ (5-II), Al_2_Zn_2_ (1-III); each of them contains only three Al–M bonds, while the Al–Al and M–M bonds are absent in these metal clusters. All these structural isomers are characterized by pronounced asymmetry, with an almost complete absence of any symmetry elements; only some of them have a plane of symmetry and a center of symmetry.

As can be seen from Figure 1, the Al–Al bond is present in the molecular structures of the most stable Al_2_M_2_ metal clusters of the first five 3*d*-elements (Sc–Mn), whereas, in the other five (Fe–Zn), it is absent. The M–M bond occurs more often: it occurs in eight out of ten metal clusters and is absent only in Al_2_Mn_2_ (1-I) and Al_2_Cu_2_ (1-I). It should be noted in this connection that, on the whole, the M–M bond in metal clusters of the Al_2_M_2_ type occurs more often than the Al–Al bond. This conclusion is quite consistent with the conclusion made in our previous article [24] devoted to heterobihexanuclear (AlFe) metal clusters because, for such metal clusters, a similar feature was noted, too. As to the “heterometallic” Al–M bonds, they, as it should be expected, are present in any of the structural isomers of each considered metal cluster. Metal-to-metal interatomic distances in any of these metal clusters in the vast majority of cases exceed 200 pm (see Table 2 and Table 3). The only exceptions here are the distances between vanadium atoms in Al_2_V_2_, that are much shorter than all other interatomic metal–metal distances (Table S1); at the same time, in all Al_2_V_2_ metal clusters, the formation of V–V chemical bonds take place, that contributes to a decrease in the above distances. In this regard, it should be noted that, according to the data of our calculation, besides Al_2_V_2_, there is only one more metal cluster of the Al_2_M_2_ type, in all structural isomers of which there is an M–M bond, and namely Al_2_Ti_2_. In any separately taken Al_2_M_2_ metal cluster, in the average statistical relation, the longest are the Al–Al chemical bonds, the shortest are the M–M bonds; Al–M bonds have an intermediate-length between them. The only exception is the Al_2_Sc_2_ metal cluster, in whose structural isomers the Sc–Sc bonds have the greatest length. These facts become quite clear if we take into account that the atomic radius of Al is 143 pm, Sc—162 pm, and the atomic radii of the remaining 3*d*-elements are in the range from 124 pm (Ni) to 147 pm (Ti). The planar angles formed by the three metal atoms are generally less than 90°; a similar situation takes place for dihedral (torsion) angles. The features just noted regarding bond lengths and angles are observed in general and for structural isomers with higher total energies compared to those for the most stable isomers, data on which are presented in Table 2 and Table 3.

As can be seen from the data in Tables S1–S10, many of the most energetically stable metal clusters, and namely Al_2_Cr_2_ (1-I), Al_2_Mn_2_ (1-I), Al_2_Ni_2_ (1-I), Al_2_Cu_2_ (1-I), and Al_2_Zn_2_ (1-I), i.e., five out of ten are low-spin and diamagnetic, high-spin are only two of them—Al_2_Fe_2_ (5-I) and Al_2_Co_2_ (5-I), and the other three occupy an intermediate position between them (Al_2_Sc_2_ (3-I), Al_2_Ti_2_ (3-I), Al_2_V_2_ (3-I)).

According to the NBO analysis data, the charges on the Al and M atoms included in the composition of studied metal clusters are, on the whole, relatively small and do not exceed 1.00 in absolute value. However, in the case of Al_2_Sc_2_, Al_2_Ti_2_, Al_2_Mn_2_, Al_2_Zn_2_, the charges on the M atoms are positive, and on the Al atoms are negative, while, in the case of the other seven metal clusters of this type, the opposite situation takes place (Table S10). Such a situation with the electron density distribution is quite understandable if we take into account that the electronegativity of Al on the Pauling scale (1.61) is greater than the electronegativity of Sc, Ti, and Mn (1.35, 1.54, and 1.55, respectively), but less than the electronegativity of V (1.63). Cr (1.66), Fe (1.83), Co (1.88), Ni (1.91), Cu (1.90). Some discrepancy between the values of the electronegativity of Al and M atoms and the distributions of charges on atoms in the corresponding metal cluster Al_2_M_2_ is found only in the case of Al_2_Zn_2_, in which the charges on the aluminum atoms are negative and the charges on the atoms are positive, although the electronegativity of Zn (1.65) is higher than the electronegativity of Al (1.61). It is noteworthy that Al_2_Zn_2_ is the only one among presented in Table 5 metal clusters, where the charges on Al atoms differ not only in magnitude, but also in sign.

As it may be seen in Figure 2 and Figure 3, the HOMO and LUMO images for different Al_2_M_2_, as well as their energies, are rather significantly different each other. Moreover, for 5 metal clusters out of 10, and namely Al_2_Sc_2_ (3-I), Al_2_Ti_2_ (3-I), Al_2_V_2_ (3-I), Al_2_Fe_2_ (5-I), and Al_2_Co_2_ (5-I), there is a rather noticeable difference even between those HOMO and LUMO, which have electrons with opposite spins (+1/2 and −1/2). At the same time, interestingly, all these metal clusters have *M_S_* > 1, while the other five, namely Al_2_Cr_2_ (1-I)*, Al_2_Mn_2_ (1-I)*, Al_2_Ni_2_ (1-I), Al_2_Cu_2_ (1-I), and Al_2_Zn_2_ (1-I)*, have *M_S_* = 1 and are low-spin (Table 5). It is noteworthy that, among these low-spin metal clusters, only two, namely Al_2_Ni_2_ (1-I), Al_2_Cu_2_ (1-I), do not contain unpaired electrons (since only for them the values <S**2> = 0.0000), while the other three—Al_2_Cr_2_ (1-I)*, Al_2_Mn_2_ (1-I)*, and Al_2_Zn_2_ (1-I)*—contain them (and, therefore, are biradicals), as evidenced by their nonzero values of operator of the square of the proper angular momentum of the total spin of the system <S**2> (Table 5). As for the distribution of spin density, in this respect, each of the metal clusters under examination gives its own individual picture, any similarities between which are not found (Figure 4).

According to results of DFT OPBE/QZVP calculation, the values of the Gibbs free energy of formation Δ_f_*G*^0^(298 K) even for the most stable Al_2_M_2_ metal clusters turn out to be positive (Table 4); that means that any of them cannot be obtained by direct interaction between *metallic* aluminum and *metallic* 3*d*-element M being in solid state. However, the situation changes radically when simple substances, formed by aluminum and 3*d*-element M, are in a *gaseous* state, i.e., by using reactions of type (1) (M is 3d-element):

2Al(gas) + 2M(gas) → Al_2_M_2_(gas). (1)

According to our calculations data, each of these gas-phase reactions is thermodynamically resolved and belongs to the number of chemical processes occurring with the so-called enthalpy factor (Table 6). In addition, as may be seen from these data, for each of such reactions, Δ_r_*H*^0^(298 K) values, as well as Δ_r_*S*^0^(298 K) values, are negative for any of the considered Al_2_M_2_. According to concepts of classical chemical thermodynamics, all reactions for which Δ_r_*H*^0^(298 K) < 0 and Δ_r_*H*^0^(298 K) < 0 are thermodynamically allowed at relatively low temperatures and forbidden at high ones. Hence, each of the reactions of type (1) is exothermic; moreover, the thermal effect of any of them is quite significant. Following the Gibbs-Helmholtz Equation (2) for the isobaric process:

Δ_r_*G*^0^(*T*) = Δ_r_*H*^0^(298 K) − *T*Δ_r_*S*^0^(298 K). (2)

Δ_r_*H*^0^(298 K) and Δ_r_*S*^0^(298 K) are the changes in enthalpy and entropy as a result of a chemical process referred to standard conditions, *T* is the process temperature in K, Δ_r_*G*^0^ (*T*) is the dependence of the Gibbs free energy on the temperature *T* and may be found at such a temperature in which one or another of the reactions (1) will not occur due to the thermodynamic prohibition. It should be noted in this connection that this parameter is neither more nor less than the temperature of the beginning reaction reverse of the corresponding reaction of type (1), i.e., the temperature of thermal destruction of a corresponding metal cluster (*T*_td_, K) or (*t*°_td_, °C) in the gas phase; the values of this parameter for each of the most energy-stable Al_2_M_2_ metal clusters under study are presented in Table 7.

As it can be seen from the table above, this temperature is very large, and, for many of these, most stable Al_2_M_2_ metal clusters exceed 2000 °C; the only exceptions against this background are Al_2_Cr_2_ (1-I), Al_2_Cu_2_ (1-I), and Al_2_Zn_2_ (1-I). However, the most stable in this respect among all considered compounds is Al_2_V_2_ (3-I), while the least stable is Al_2_Zn_2_ (1-I).

## 4. Calculation Method

The quantum-chemical calculations of Al_2_Sc_2_, Al_2_Ti_2_, Al_2_V_2_, Al_2_Cr_2_, Al_2_Mn_2_, Al_2_Fe_2_, Al_2_Co_2_, Al_2_Ni_2_, Al_2_Cu_2_, and Al_2_Zn_2_ metal clusters under study were done using the density functional theory (DFT) combining the standard extended split-valence QZVP basis [27,28] and the OPBE functional [29,30]. According to the results that were published in References [27,28,29,30,31,32,33,34], the given method allows to obtain the most accurate estimation of ratio between energies of the high-spin state and low-spin state and, at the same time, rather reliably predicts the paramount geometric parameters of molecular structures for various compounds containing 3*p*- and 3*d*-element atoms. Quantum-chemical models of the molecular structures, of each of the Al_2_M_2_ metal clusters, were built with using GAUSSIAN09 software [35]. As in our earlier publications [16,17,18,19,20,21,22,23,24], the accordance of the found stationary points to the fact that the energy minima was confirmed by calculation of the second derivatives with respect to the Cartesian coordinates of atoms. Whereinto all equilibrium structures corresponding to the minima at the potential energy surface revealed only real positive frequency values, the parameters of the molecular structures, for spin multiplicities (*M_S_*) more than 1, were determined using the so-called unrestricted method (***UOPBE***), and the ones for *M_S_* = 1 were determined using the so-called restricted method (***ROPBE***). In those cases when *M_S_* was equal to 1, the unrestricted method in conjunction with the GUESS = Mix option was also used. NBO analysis of the metal clusters under consideration (Natural Population, Natural Electron Configurations, and Natural Atomic Orbital Occupancies) was carried out according to the procedure described in the works of References [36,37]. NBO 3.0 version built-in GAUSSIAN09 was used. The energetically most favorable structure has always been checked with the STABLE = OPT procedure; in all cases, the wave function corresponding to it was stable. The standard thermodynamic parameters of formation of metal clusters considered here, and namely standard enthalpy Δ_f_*H*^0^(298 K), entropy S_f_^0^(298K), and Gibbs’ energy Δ_f_*G*^0^(298 K) of formation for these metal clusters, were calculated using the method described in Reference [38].

## 5. Conclusions

As can be seen from the data presented above, most of the Al_2_M_2_ metal clusters of 3*d*-elements considered by us, despite their very simple stoichiometric composition, are, nevertheless, capable of forming a rather significant amount (as a rule, more than 10) of structural isomers with different geometric shapes and key parameters of molecular structures as well as with various the spin multiplicities of the ground states. The amount of these isomers for a concrete Al_2_M_2_ metal cluster substantially depends on the nature of the atoms of the 3*d*-element M included in its composition; at the same time, that is characteristic, and no correlation between this same amount and the electronic configuration of the corresponding 3*d*-element are obtained. Nevertheless, in the Sc-Zn series, there is a rather distinct increase in the amount of planar molecular structures in comparison with three-dimensional ones (tetrahedral and/or trigonal pyramidal). It is characteristic that all structural isomers of Al_2_M_2_ revealed as a result of our calculations are either completely asymmetric or have a maximum of three symmetry elements—one axis of symmetry of the second order and two planes of symmetry (as is the case, for example, in Al_2_Sc_2_ (5-II), Al_2_Cr_2_ (1-III), or Al_2_Cu_2_ (5-V)) or one axis of symmetry of the second order, one plane of symmetry and center of symmetry (for example, in Al_2_Cu_2_ (5-I), Al_2_Cu_2_ (5-IV) or Al_2_Zn_2_ (3-II)). In this connection, that attention is drawn to the interesting fact that central-symmetric structural isomers are observed only among the Al_2_Cu_2_ and Al_2_Zn_2_ metal clusters (see Supplementary Materials, Figures S9 and S10). In the same series, the tendency towards a gradual decrease in the amount of structural isomers with the Al–Al bond is quite clearly expressed; if, in the case of Al_2_Sc_2_ and Al_2_Ti_2_, such a bond is absent only in one of 7 and 13 of their isomers (Al_2_Sc_2_ (5-II) and Al_2_Ti_2_ (3-II), respectively), then, in the case of Al_2_Cu_2_ and Al_2_Zn_2_, this bond is present only in one of 12 and 8 isomers (Al_2_Cu_2_ (5-V) and Al_2_Zn_2_ (5-I), respectively), and, among the Al_2_Ni_2_ isomers, there is not one in which there was an Al–Al bond. Be that as it may, all the most stable Al_2_M_2_ metal clusters shown in Figure 1 are in fact distorted tetrahedra. This seems to be quite natural since, in the presence of two different chemical elements in each of them, there is a violation of the symmetry, wherein *T_d_* is broken towards structures with symmetry *C_2v_* and *C_s_*, with all possible alternations of two types of atoms. As can be seen from the data presented in the Table 6 and Table 7, all the most stable Al_2_M_2_ metal clusters are characterized by very high thermal stability, and this is despite the fact that, for any of them, the values are Δ_f_*G*^0^(298K) > 0 (Table 4), and, therefore, none of them can be obtained directly from metallic aluminum and of the metal that is formed by the 3*d*-element M. This circumstance allows us to assert that, if these metal clusters appear in some way in the experiment, then, they will be sufficiently “viable” for independent (separated, solo) existence.

Speaking about the possible practical application of metal clusters of this type, it should be noted that the most promising here is their use for the creation of new composite materials and alloys based on polymetallic nanoparticles, as well as alloying and doping with them of traditional alloys based on both ferrous and non-ferrous metals. It is not excluded, and even quite possible, that at least some of these alloys will possess very exotic physical and mechanical properties. They are also very promising as potential quantum dots, the possibilities of technologies with the use of which are far from being exhausted; possible areas of their use can also be catalysis, creation of specific electrochemical systems, and semiconductors.

## Figures and Tables

**Table 1:** Total number of Al_2_M_2_ (*N*) metal clusters (M–3*d*-element).

M	Sc	Ti	V	Cr	Mn	Fe	Co	Ni	Cu	Zn
*N*	7	13	14	9	20	22	21	6	12	8

**Table 2:** Structural parameters of most stable Al_2_M_2_ metal clusters (M = Sc, Ti, V, Cr, Mn).

	Al_2_Sc_2_ (3-I)	Al_2_Ti_2_ (3-I)	Al_2_V_2_ (3-I)	Al_2_Cr_2_ (1-I)*	Al_2_Mn_2_ (1-I)*
All distances between metal atoms, *pm*					
Al1Al2	254.5	249.3	252.8	248.8	257.3
Al1M1	272.8	263.7	257.9	266.2	257.6
Al1M2	272.8	263.8	257.9	266.3	257.6
Al2M1	272.8	263.7	257.6	266.4	257.6
Al2M2	272.8	263.6	257.6	266.2	257.7
M1M2	277.7	210.3	170.8	259.6	280.0
All plane angles between metal atoms, *deg*					
Al1Al2M1	62.2	61.8	60.7	62.1	60.0
Al1Al2M2	62.2	61.8	60.7	62.2	60.0
Al2Al1M1	62.2	61.8	60.6	62.2	60.0
Al2Al1M2	62.2	61.7	60.6	62.1	60.0
Al1M1Al2	55.6	56.4	58.7	55.7	59.9
Al1M1M2	59.4	66.5	70.7	60.8	57.1
M1Al1M2	61.2	47.1	38.7	58.3	65.8
Al1M2Al2	55.6	56.4	58.7	55.7	59.9
Al1M2M1	59.4	66.4	70.7	60.8	57.1
Al2M1M2	59.4	66.4	70.7	60.8	57.1
M1Al2M2	61.2	47.1	38.7	58.3	65.8
Al2M2M1	59.4	66.5	70.6	60.9	57.1
Selected torsion angles between metal atoms, *deg*					
Al1Al2M1M2	−71.8	−76.5	−78.6	72.9	−68.1
Al1Al2M2M1	71.8	76.4	78.6	−72.8	68.1
Al2Al1M1M2	71.8	76.4	78.6	−72.8	68.1
Al2Al1M2M1	−71.8	−76.5	−78.6	72.9	−68.0
M1Al1Al2M2	−70.3	−54.0	−44.7	66.9	−77.7
M1Al2Al1M2	70.3	54.0	44.7	−66.9	77.7
Al1M1M2Al2	−65.6	−62.1	−62.6	64.7	−73.0
Al1M2M1Al2	65.6	62.1	62.6	−64.7	73.0
Al1M1Al2M2	71.8	76.5	78.6	−72.9	68.1
Al1M2Al2M1	−71.8	−76.4	−78.6	72.8	−68.1
Al2M1Al1M2	−71.8	−76.4	−78.6	72.9	−68.1
Al2M2Al1M1	71.8	76.5	78.6	−729.8	68.1

**Table 3:** Structural parameters of most stable Al_2_M_2_ metal clusters (M = Fe, Co, Ni, Cu, Zn).

	Al_2_Fe_2_ (5-I)	Al_2_Co_2_ (5-I)	Al_2_Ni_2_ (1-I)	Al_2_Cu_2_ (1-I)	Al_2_Zn_2_ (1-I)*
All distances between metal atoms, *pm*					
Al1Al2	259.3	256.6	262.2	250.5	276.9
Al1M1	249.8	243.5	233.6	242.8	261.0
Al1M2	249.6	243.4	233.6	243.0	261.0
Al2M1	249.8	243.3	233.6	243.0	261.0
Al2M2	249.6	243.3	233.6	243.1	261.0
M1M2	198.6	209.0	220.0	254.7	260.2
All plane angles between metal atoms, *deg*					
Al1Al2M1	58.7	58.2	55.9	58.9	58.0
Al1Al2M2	58.7	58.2	55.9	59.0	58.0
Al2Al1M1	58.7	58.2	55.9	59.0	58.0
Al2Al1M2	58.7	58.2	55.9	59.0	58.0
Al1M1Al2	62.5	63.6	68.3	62.1	64.1
Al1M1M2	66.5	64.6	61.9	58.4	60.1
M1Al1M2	46.9	50.8	56.2	63.2	59.8
Al1M2Al2	62.5	63.6	68.3	62.0	64.1
Al1M2M1	66.6	64.6	61.9	58.3	60.1
Al2M1M2	66.5	64.6	61.9	58.4	60.1
M1Al2M2	46.9	50.9	56.2	63.2	59.8
Al2M2M1	66.6	64.6	61.9	58.4	60.1
Selected torsion angles between metal atoms, *deg*					
Al1Al2M1M2	74.7	−72.8	68.8	68.3	68.9
Al1Al2M2M1	−74.8	72.9	−68.8	−68.2	−68.9
Al2Al1M1M2	−74.7	72.8	−68.8	−68.3	−68.9
Al2Al1M2M1	74.8	−72.8	68.8	68.3	68.9
M1Al1Al2M2	55.5	−60.7	69.4	75.4	72.1
M1Al2Al1M2	−55.5	60.7	−69.4	−75.4	−72.1
Al1M1M2Al2	68.9	−71.4	79.0	74.5	75.5
Al1M2M1Al2	−68.9	71.4	−79.0	−74.5	−75.5
Al1M1Al2M2	−74.7	72.8	−68.8	−68.3	−68.9
Al1M2Al2M1	74.8	−72.9	68.8	68.2	68.9
Al2M1Al1M2	74.7	−72.8	68.8	68.3	68.9
Al2M2Al1M1	−74.8	72.8	−68.8	−68.3	−68.9

**Table 4:** Standard thermodynamic parameters of formation for the most energetically stable Al_2_M_2_ metal clusters (M–3*d*-element).

Metal Cluster	Δ_f_*H*^0^(298 K) kJ/mol	*S_f_*^0^(298 K) J/mol K	Δ_f_*G*^0^(298 K), kJ/mol
Al_2_Sc_2_ (3-I)	677.9	387.8	599.9
Al_2_Ti_2_ (3-I)	833.4	375.9	756.5
Al_2_V_2_ (3-I)	521.0	378.5	439.2
Al_2_Cr_2_ (1-I)*	923.0	361.6	848.1
Al_2_Mn_2_ (1-I)*	469.8	362.2	397.7
Al_2_Fe_2_ (5-I)	691.7	387.4	609.4
Al_2_Co_2_ (5-I)	701.8	380.5	623.3
Al_2_Ni_2_ (1-I)	669.8	375.6	592.5
Al_2_Cu_2_ (1-I)	662.7	358.9	592.5
Al_2_Zn_2_ (1-I)*	604.7	372.9	535.4

**Table 5:** Charge distribution (in units of electron charge) on various Al and M atoms and the values of operator of the square of the proper angular momentum of the total spin of the system <S**2> for most stable Al_2_M_2_ metal clusters according to NBO analysis data (M−3*d*-element).

Charges on the Al and M Atoms in the Al_2_M_2_ Metal Cluster, ē				Charges on the Al and M Atoms in the Al_2_M_2_ Metal Cluster, ē			
Al_2_Sc_2_ (3-I) (<S**2> = 2.0534)				Al_2_Fe_2_ (5-I) (<S**2> = 6.7424)			
Al1	Al2	Sc1	Sc2	Al1	Al2	Fe1	Fe2
−0.0493	−0.0494	+0.0492	+0.0495	+0.2022	+0.2020	−0.1996	−0.2048
Al_2_Ti_2_ (3-I) (<S**2> = 2.2578)				Al_2_Co_2_ (5-I) (<S**2> = 6.2633)			
Al1	Al2	Ti1	Ti2	Al1	Al2	Co1	Co2
+0.1420	+0.1416	−0.1416	−0.1420	+0.2443	+0.2439	−0.2436	−0.2446
Al_2_V_2_ (3-I) (<S**2> = 2.0204)				Al_2_Ni_2_ (1-I) (<S**2> = 0.0000)			
Al1	Al2	V1	V2	Al1	Al2	Ni1	Ni2
+0.1607	+0.1599	−0.1606	−0.1600	+0.3525	+0.3525	−0.3525	−0.3525
Al_2_Cr_2_ (1-I)* (<S**2> = 4.6791)				Al_2_Cu_2_ (1-I) (<S**2> = 0.0000)			
Al1	Al2	Cr1	Cr2	Al1	Al2	Cu1	Cu2
−0.0315	−0.0317	+0.0312	+0.0320	+0.1116	+0.1127	−0.1128	−0.1115
Al_2_Mn_2_ (1-I)* (<S**2> = 4.7528)				Al_2_Zn_2_ (1-I)* (<S**2> = 0.4335)			
Al1	Al2	Mn1	Mn2	Al1	Al2	Zn1	Zn2
−0.1351	−0.1344	+0.1347	+0.1348	−0.2163	+0.1179	+0.0492	+0.0492

**Table 6:** Thermodynamic parameters Δ_r_*H*^0^(298 K) and Δ_r_*S*^0^(298 K) for the reactions (1–10) according to DFT OPBE/QZVP method.

Metal Cluster	Δ_r_*H*^0^(298 K), kJ	Δ_r_*S*^0^(298 K), J/K	Metal Cluster	Δ_r_*H*^0^(298 K), kJ	Δ_r_*S*^0^(298 K), J/K
Al_2_Sc_2_ (3-I)	−738.5	−290.4	Al_2_Fe_2_ (5-I)	−799.5	−302.3
Al_2_Ti_2_ (3-I)	−772.0	−313.4	Al_2_Co_2_ (5-I)	−805.7	−307.2
Al_2_V_2_ (3-I)	−1167.9	−315.2	Al_2_Ni_2_ (1-I)	−847.6	−317.5
Al_2_Cr_2_ (1-I)	−528.3	−315.7	Al_2_Cu_2_ (1-I)	−672.1	−302.5
Al_2_Mn_2_(1-I)	−749.6	−313.9	Al_2_Zn_2_ (1-I)	−315.0	−278.2

**Table 7:** Temperatures of the beginning thermal destruction (*T*_td_, K) and (*t*°_td_, °C) of the most energetically stable Al_2_M_2_ metal clusters.

Metal Cluster	*T*_td_, K	*t*°_td_, °C	Metal Cluster	*T*_td_, K	*t*°_td_, °C
Al_2_Sc_2_ (3-I)	2543.0	2269.8	Al_2_Fe_2_ (5-I)	2644.7	2371.5
Al_2_Ti_2_ (3-I)	2463.3	2190.1	Al_2_Co_2_ (5-I)	2622.7	2349.5
Al_2_V_2_ (3-I)	3705.3	3432.1	Al_2_Ni_2_ (1-I)	2669.6	2396.4
Al_2_Cr_2_ (1-I)	1673.4	1400.2	Al_2_Cu_2_ (1-I)	2221.8	1948.6
Al_2_Mn_2_ (1-I)	2388.0	2114.8	Al_2_Zn_2_ (1-I)	1132.3	859.1

## Data Availability

Data sharing is not applicable.

## Supplementary Materials

The following are available online at https://www.mdpi.com/article/10.3390/ma14226836/s1, Figure S1: Molecular structures of Al_2_Sc_2_ metal clusters, Figure S2: Molecular structures of Al_2_Ti_2_ metal clusters, Figure S3: Molecular structures of Al_2_V_2_ metal clusters, Figure S4: Molecular structures of Al_2_Cr_2_ metal clusters, Figure S5: Molecular structures of Al_2_Mn_2_ metal clusters, Figure S6: Molecular structures of Al_2_Fe_2_ metal clusters, Figure S7: Molecular structures of Al_2_Co_2_ metal clusters, Figure S8: Molecular structures of Al_2_Ni_2_ metal clusters, Figure S9: Molecular structures of Al_2_Cu_2_ metal clusters, Figure S10: Molecular structures of Al_2_Zn_2_ metal clusters. Table S1: Relative energies and spin multiplicities of the ground states of various isomers of Al_2_Sc_2_ metal clusters, Table S2: Relative energies and spin multiplicities of the ground states of various isomers of Al_2_Ti_2_ metal clusters, Table S3: Relative energies and spin multiplicities of the ground states of various isomers of Al_2_V_2_ metal clusters, Table S4: Relative energies and spin multiplicities of the ground states of various isomers of Al_2_Cr_2_ metal clusters, Table S5: Relative energies and spin multiplicities of the ground states of various isomers of Al_2_Mn_2_ metal clusters, Table S6: Relative energies and spin multiplicities of the ground states of various isomers of Al_2_Fe_2_ metal clusters, Table S7: Relative energies and spin multiplicities of the ground states of various isomers of Al_2_Co_2_ metal clusters, Table S8: Relative energies and spin multiplicities of the ground states of various isomers of Al_2_Ni_2_ metal clusters, Table S9: Relative energies and spin multiplicities of the ground states of various isomers of Al_2_Cu_2_ metal clusters, Table S10: Relative energies and spin multiplicities of the ground states of various isomers of Al_2_Zn_2_ metal clusters, Table S11: The values of energies (are given in eV) of highest occupied (HOMO) and lowest unoccupied (LUMO) molecular orbitals, and values of gap. The symbol “a” corresponds to electron with spin (+1/2), “b”, to electron with spin (–l/2).
