# Supplementary material for: Quantum-Chemical Consideration of Al2M2 Tetranuclear Metal Clusters (M–3d-Element): Molecular/Electronic Structures and Thermodynamics
Source: Materials (Basel). 2021 Nov 12;14(22):6836. doi: 10.3390/ma14226836 (PMC8619736; doi:10.3390/ma14226836)
Supplement: Supplementary file 1 [file materials-14-06836-s001.zip › materials-1424069-supplementary.pdf]

materials

Supplementary Materials

Quantum-Chemical Consideration of Al_2M_2 Tetranuclear Metal Clusters (M–3*d*-Element): Molecular/Electronic Structures and Thermodynamics

Oleg V. Mikhailov [1,*] and Denis V. Chachkov [2]

[1] Department of Analytical Chemistry, Certification and Quality Management, Kazan National Research Technological University, K. Marx Street 68, 420015 Kazan, Russia
[2] Kazan Department of Joint Supercomputer Center of Russian Academy of Sciences—Branch of Federal Scientific Center "Scientific Research Institute for System Analysis of the RAS", Lobachevskii Street 2/31, 420111 Kazan, Russia; de2005c@gmail.com
* Correspondence: olegmkhlv@gmail.com

Citation: Mikhailov, O.V.; Chachkov, D.V. Quantum-Chemical Consideration of Al_2M_2 Tetranuclear Metal Clusters (M–3*d*-Element): Molecular/Electronic Structures and Thermodynamics. *Materials* **2021**, *14*, 6836. https://doi.org/10.3390/ma14226836

Academic Editors: Yong-Cheng Lin, Zhe Zhang, Xin-Yun Wang and Guo-Qun Zhao

Received: 30 September 2021
Accepted: 10 November 2021
Published:

Publisher's Note: MDPI stays neutral with regard to jurisdictional claims in published maps and institutional affiliations.

Copyright: © 2021 by the authors. Licensee MDPI, Basel, Switzerland. This article is an open access article distributed under the terms and conditions of the Creative Commons Attribution (CC BY) license (http://creativecommons.org/licenses/by/4.0/).

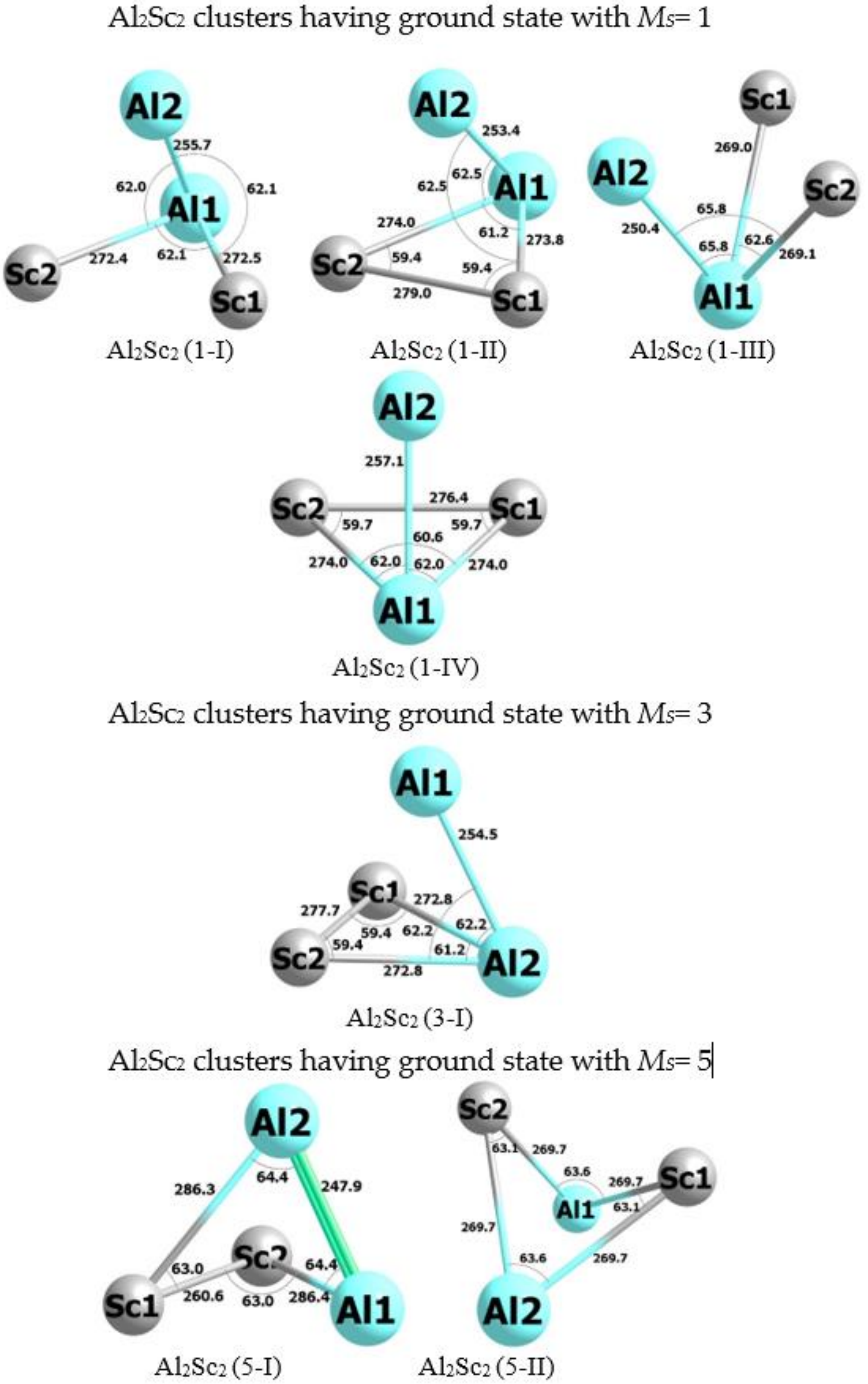

Figure S1. Molecular structures of Al_2Sc_2 metal clusters.

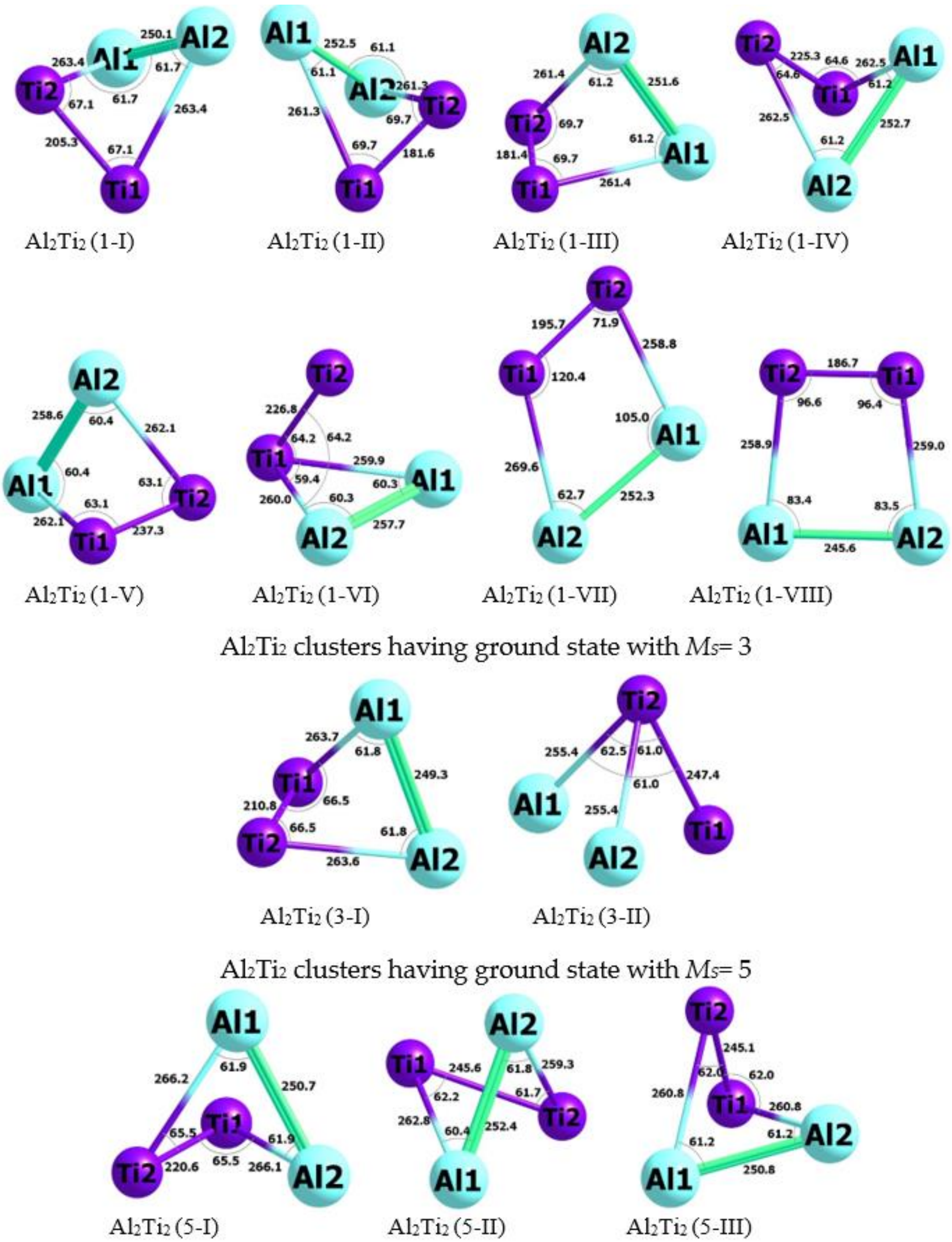

Figure S2. Molecular structures of Al_2Ti_2 metal clusters.

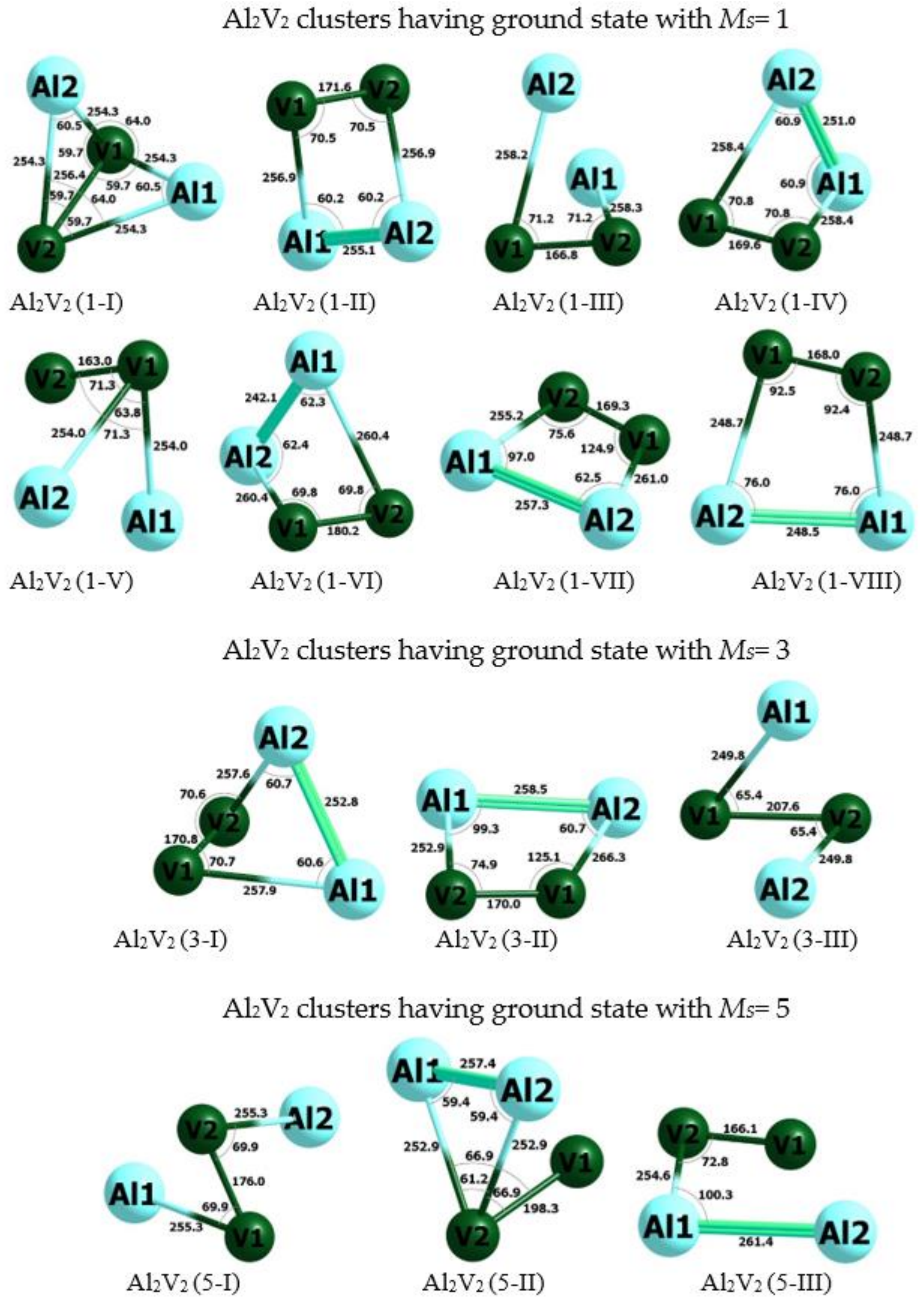

Figure S3. Molecular structures of Al_2V_2 metal clusters.

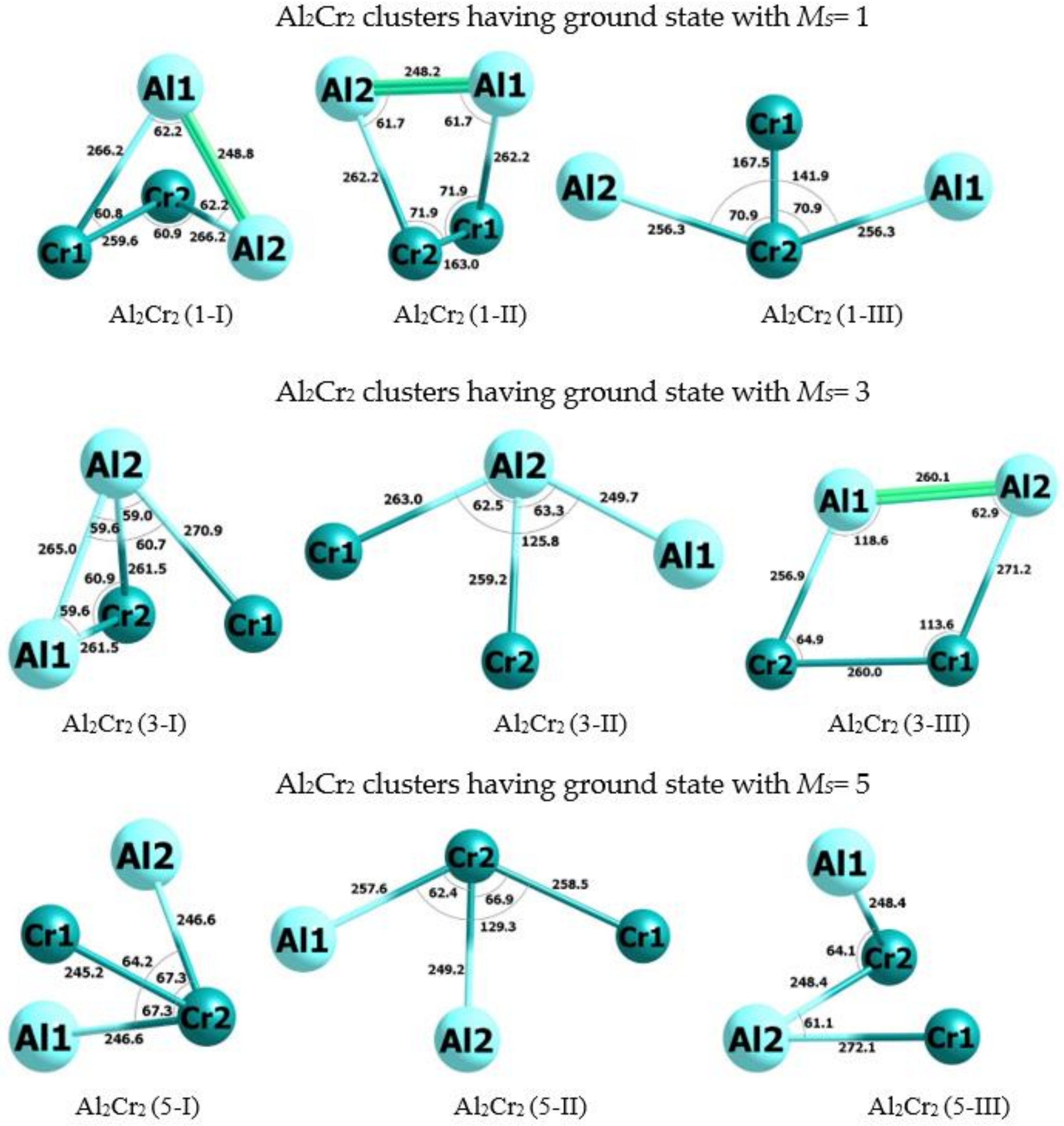

Figure S4. Molecular structures of Al_2Cr_2 metal clusters.

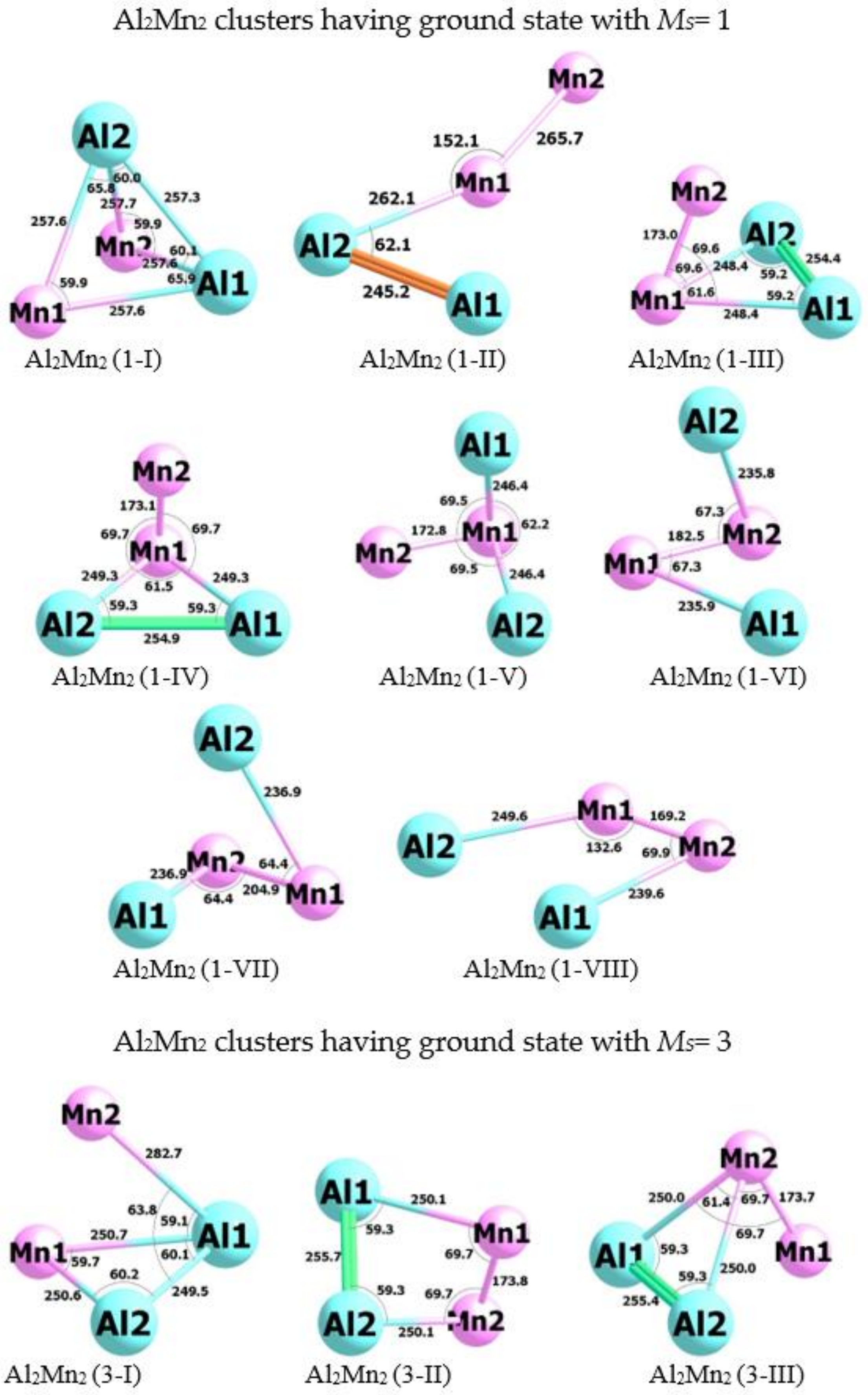

Al2Mn2 clusters having ground state with Ms= 1
Al2 Mn2 Mn1 Al1 257.6 257.3 257.7 257.6 59.9 65.8 60.0 59.9 60.1 65.9
Al2Mn2 (1-I)
Mn2 Mn1 Al2 Al1 152.1 265.7 262.1 62.1 245.2
Al2Mn2 (1-II)
Mn2 Al2 Mn1 Al1 173.0 69.6 69.6 248.4 61.6 59.2 254.4 59.2 248.4
Al2Mn2 (1-III)
Mn2 Mn1 Al2 Al1 173.1 69.7 69.7 249.3 249.3 61.5 59.3 59.3 254.9
Al2Mn2 (1-IV)
Al1 Mn1 Mn2 Al2 246.4 69.5 172.8 62.2 69.5 246.4
Al2Mn2 (1-V)
Al2 Mn2 Mn1 Al1 235.8 67.3 182.5 67.3 235.9
Al2Mn2 (1-VI)
Al2 Mn2 Mn1 Al1 236.9 236.9 64.4 64.4 204.9
Al2Mn2 (1-VII)
Al2 Mn1 Mn2 Al1 249.6 169.2 132.6 69.9 239.6
Al2Mn2 (1-VIII)
Al2Mn2 clusters having ground state with Ms= 3
Mn2 Mn1 Al1 Al2 282.7 63.8 59.1 250.7 60.1 59.7 60.2 250.6 249.5
Al2Mn2 (3-I)
Al1 Mn1 Al2 Mn2 250.1 59.3 255.7 69.7 173.8 59.3 69.7 250.1
Al2Mn2 (3-II)
Mn2 Al1 Mn1 Al2 250.0 61.4 69.7 173.7 69.7 59.3 59.3 250.0 255.4
Al2Mn2 (3-III)

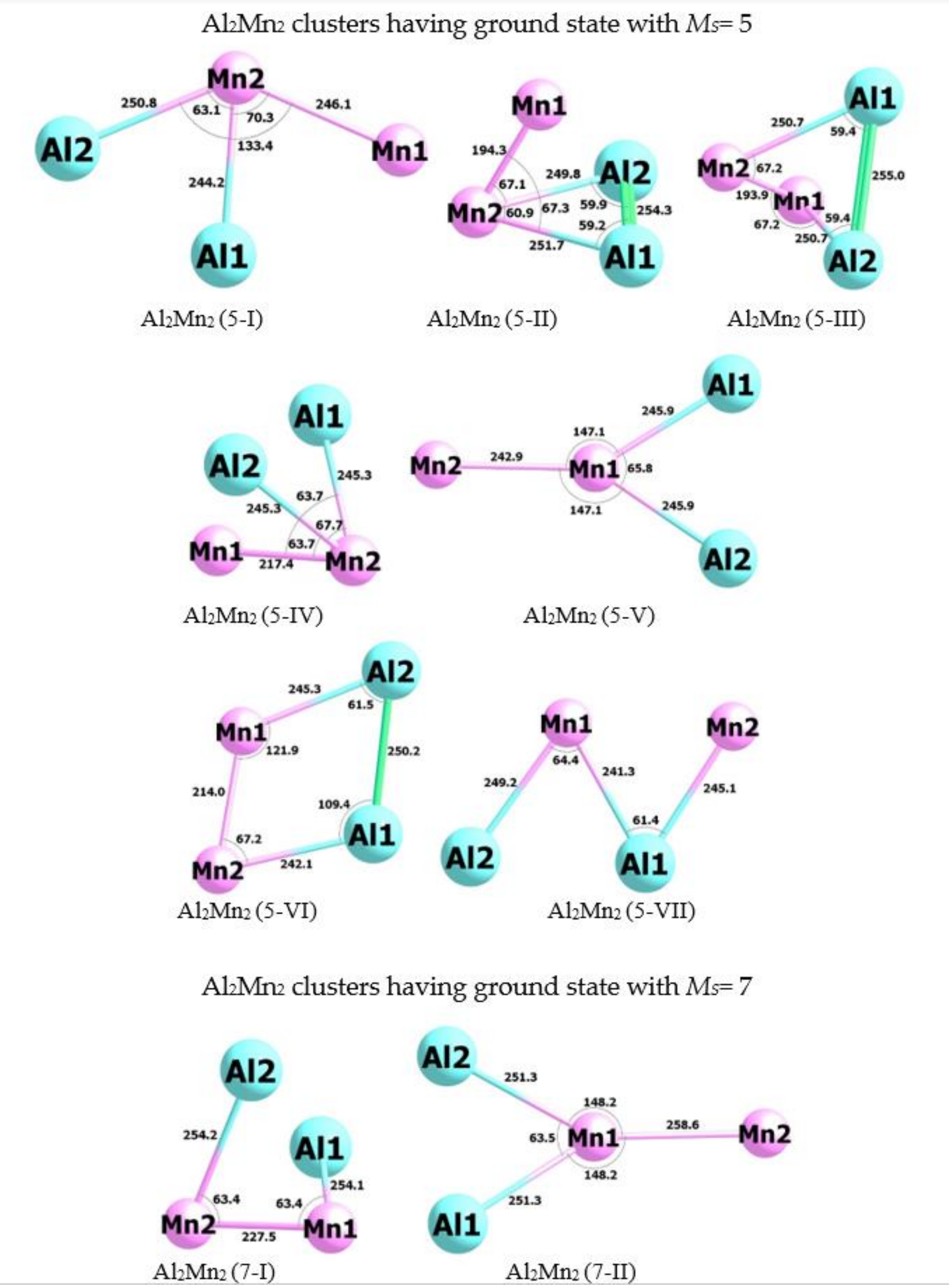

Figure S5. Molecular structures of Al_2Mn_2 metal clusters.

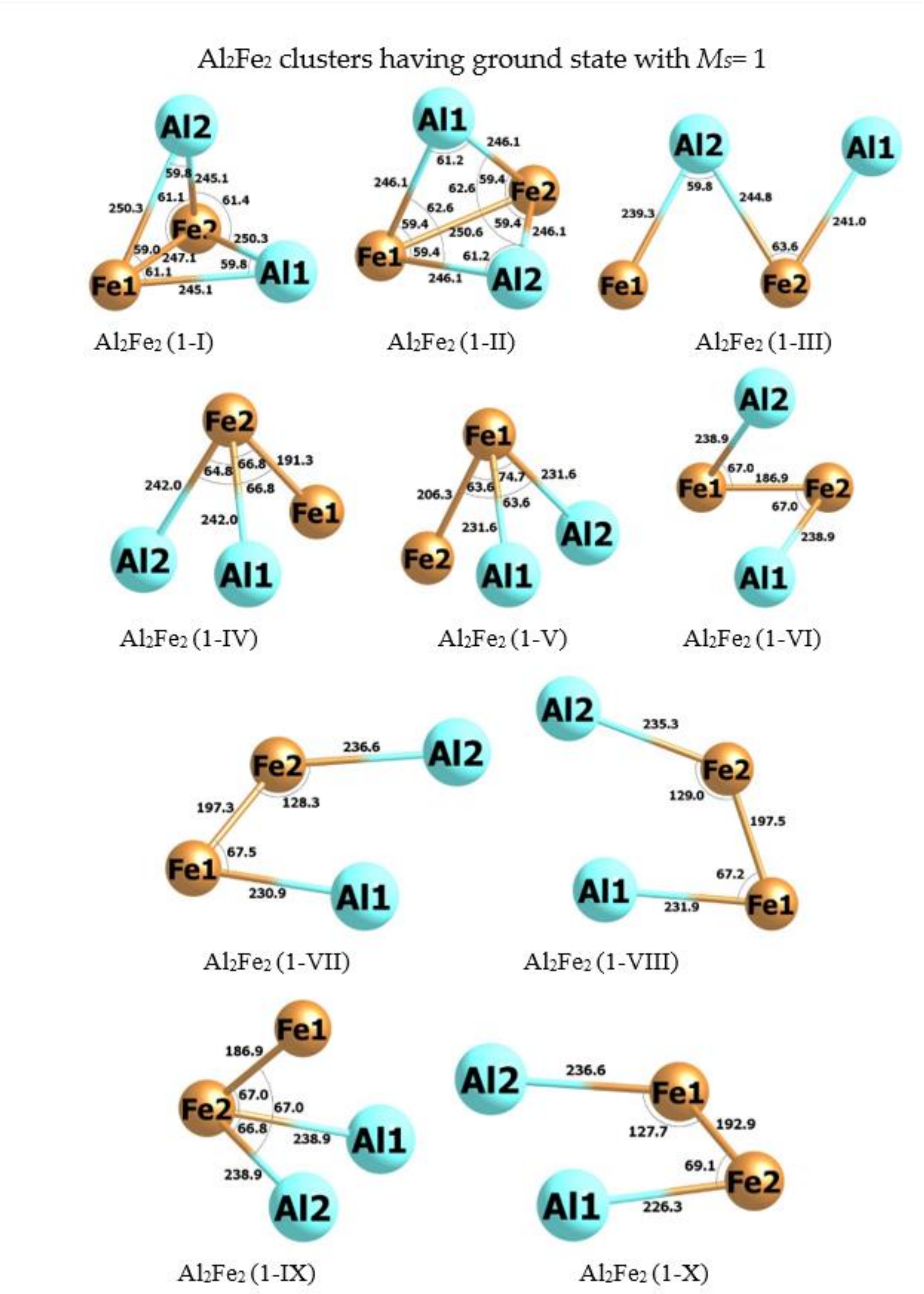
Al_2Fe_2 clusters having ground state with M_S= 1
Al_2Fe_2 (1-I)
Al_2Fe_2 (1-II)
Al_2Fe_2 (1-III)
Al_2Fe_2 (1-IV)
Al_2Fe_2 (1-V)
Al_2Fe_2 (1-VI)
Al_2Fe_2 (1-VII)
Al_2Fe_2 (1-VIII)
Al_2Fe_2 (1-IX)
Al_2Fe_2 (1-X)

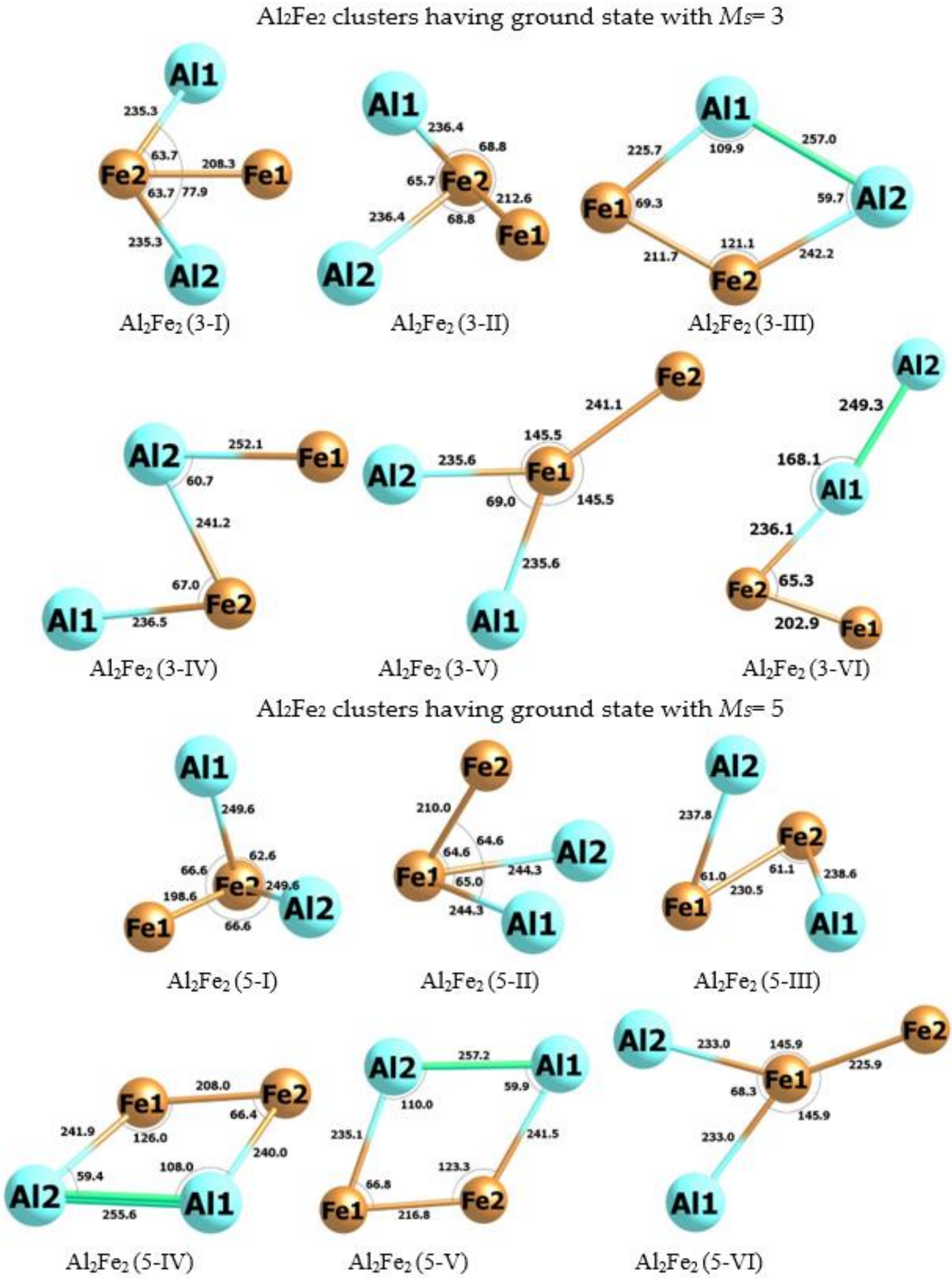

Figure S6. Molecular structures of Al_2Fe_2 metal clusters.

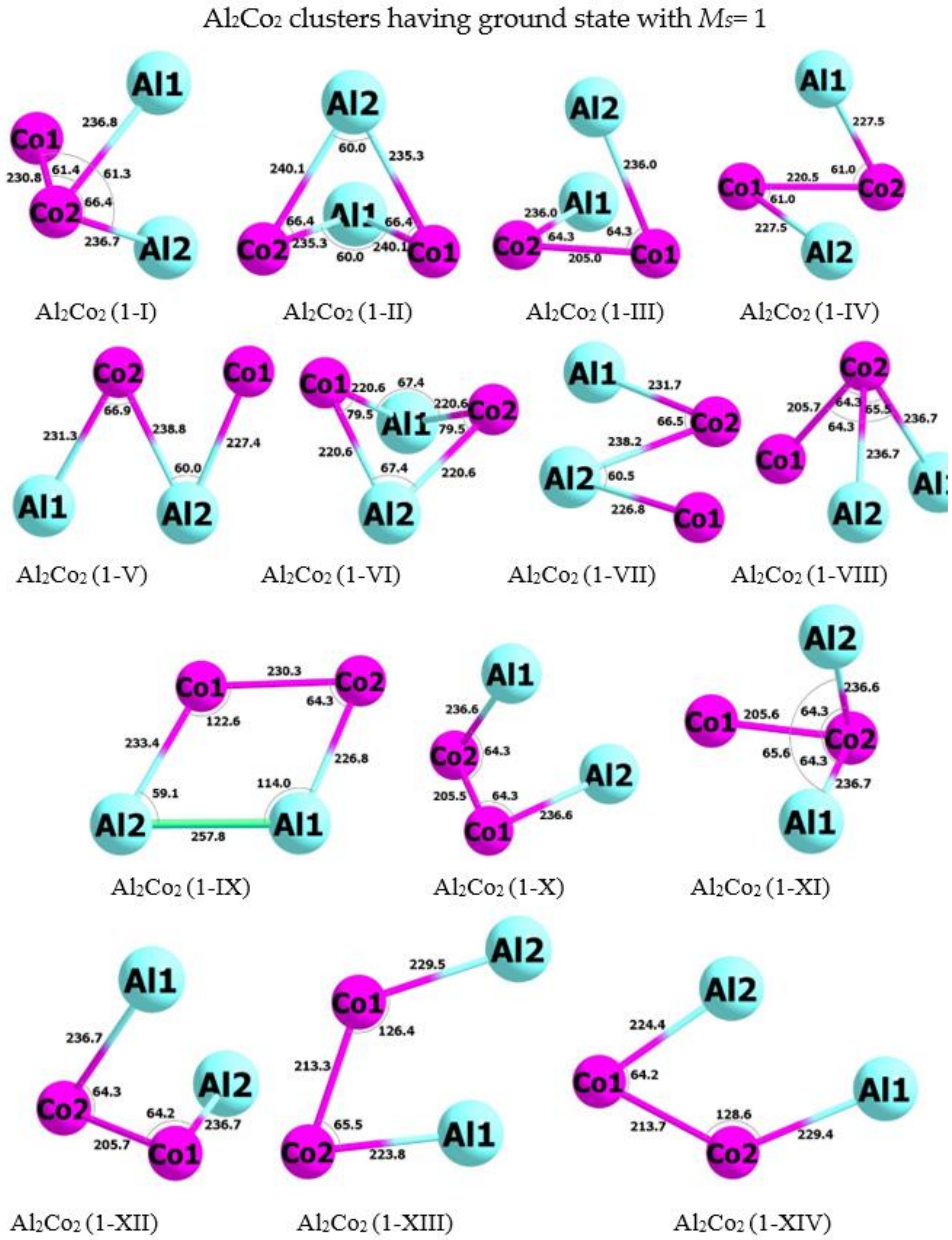
Al2Co2 clusters having ground state with Ms= 1
Al1 Co1 Co2 Al2 236.8 61.4 61.3 230.8 66.4 236.7
Al2Co2 (1-I)
Al2 Al1 Co2 Co1 60.0 240.1 235.3 66.4 66.4 235.3 60.0 240.1
Al2Co2 (1-II)
Al2 Al1 Co2 Co1 236.0 236.0 64.3 64.3 205.0
Al2Co2 (1-III)
Al1 Co1 Co2 Al2 227.5 220.5 61.0 61.0 227.5
Al2Co2 (1-IV)
Co2 Co1 Al1 Al2 66.9 231.3 238.8 227.4 60.0
Al2Co2 (1-V)
Co1 Al1 Co2 Al2 220.6 67.4 220.6 79.5 79.5 220.6 67.4 220.6
Al2Co2 (1-VI)
Al1 Co2 Al2 Co1 231.7 66.5 238.2 60.5 226.8
Al2Co2 (1-VII)
Co2 Co1 Al2 205.7 64.3 65.5 236.7 64.3 236.7
Al2Co2 (1-VIII)
Co1 Co2 Al2 Al1 230.3 64.3 122.6 233.4 226.8 114.0 59.1 257.8
Al2Co2 (1-IX)
Al1 Co2 Al2 Co1 236.6 64.3 205.5 64.3 236.6
Al2Co2 (1-X)
Al2 Co1 Co2 Al1 236.6 205.6 64.3 65.6 64.3 236.7
Al2Co2 (1-XI)
Al1 Co2 Al2 Co1 236.7 64.3 64.2 236.7 205.7
Al2Co2 (1-XII)
Al2 Co1 Co2 Al1 229.5 126.4 213.3 65.5 223.8
Al2Co2 (1-XIII)
Al2 Co1 Co2 Al1 224.4 64.2 213.7 128.6 229.4
Al2Co2 (1-XIV)

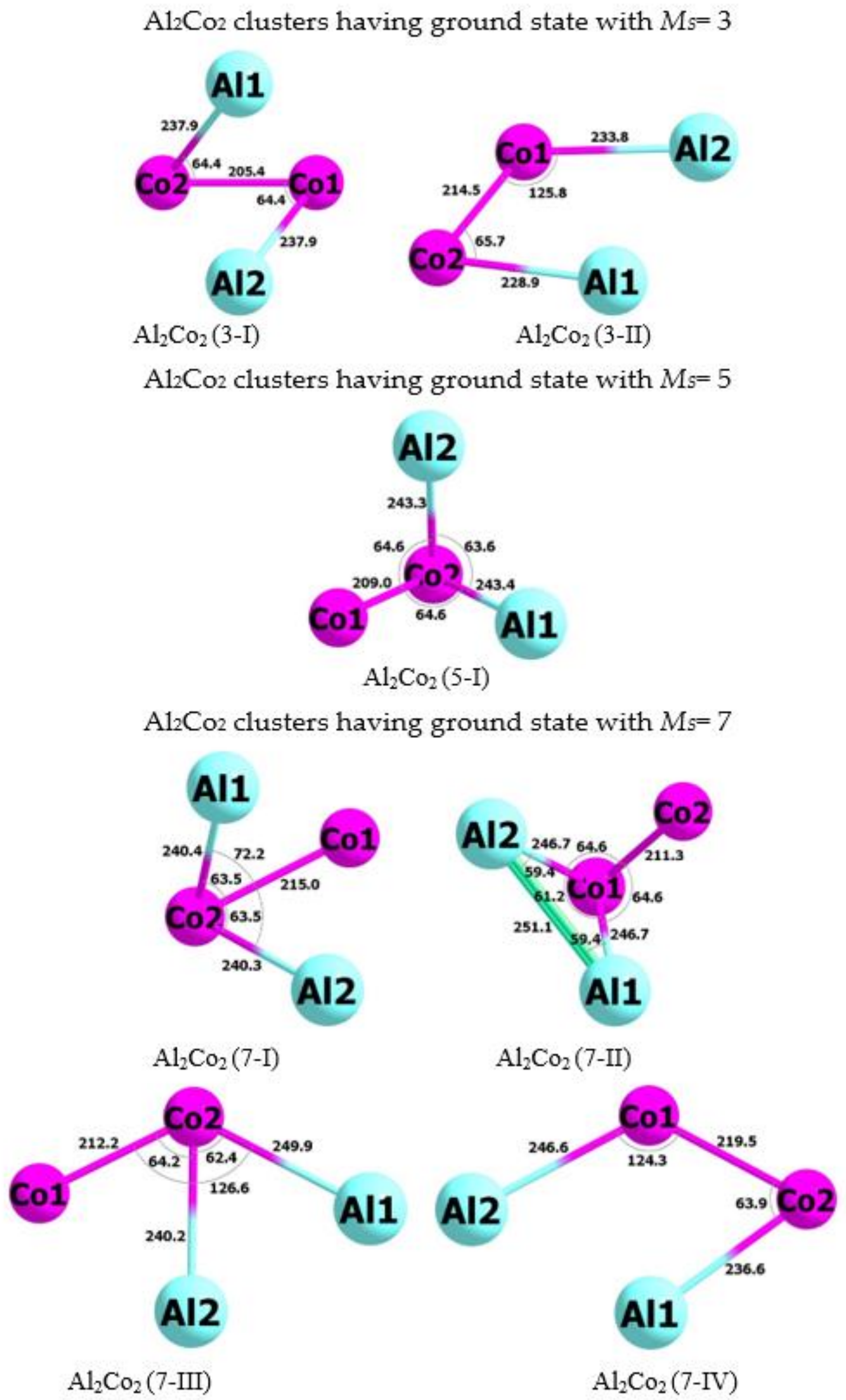

Figure S7. Molecular structures of Al_2Co_2 metal clusters.

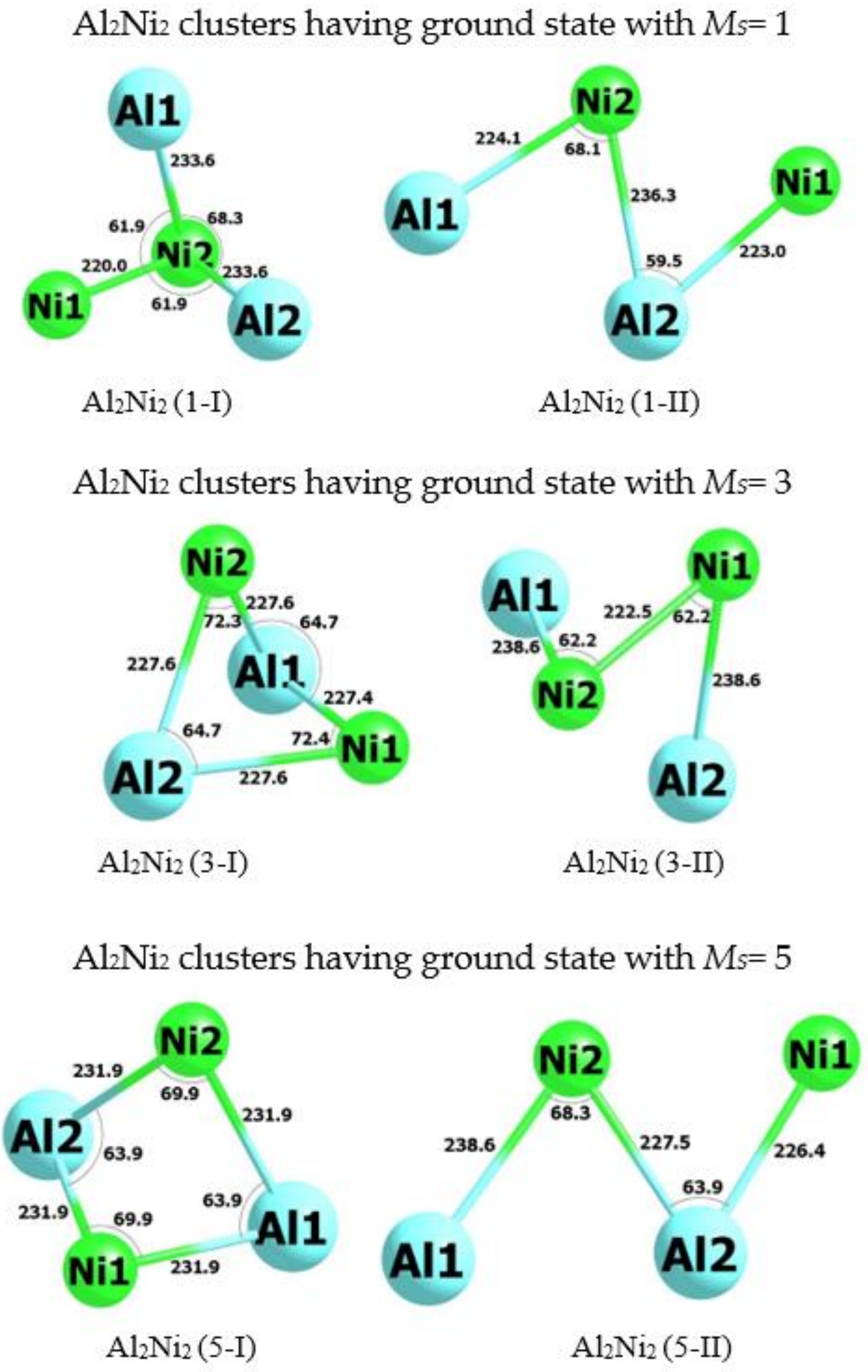

Figure S8. Molecular structures of Al_2Ni_2 metal clusters.

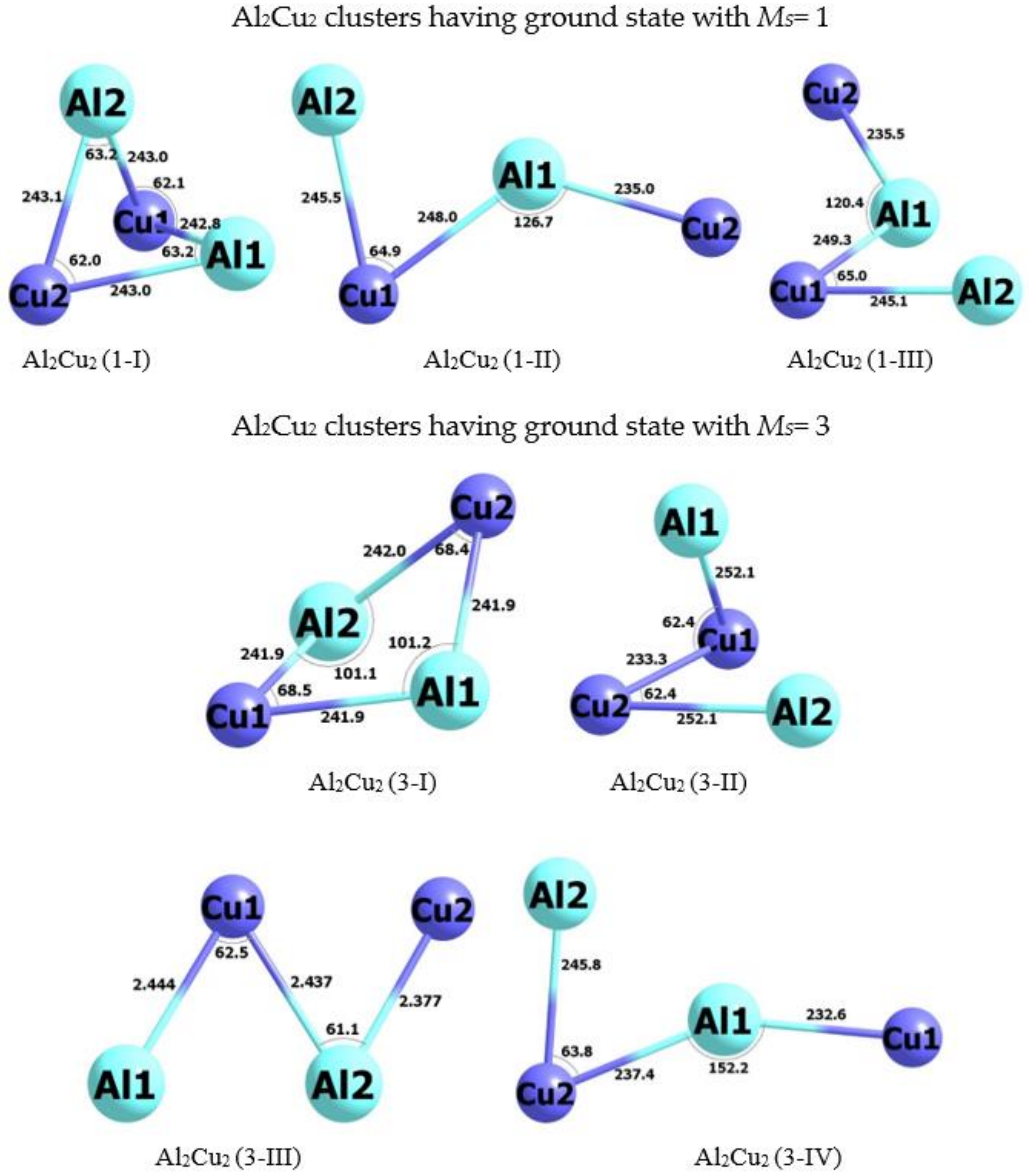
Al2Cu2 clusters having ground state with Ms= 1
Al2
Cu1
Al1
Cu2
63.2
243.0
62.1
243.1
242.8
63.2
62.0
243.0
Al2Cu2 (1-I)
245.5
248.0
64.9
235.0
126.7
Al2Cu2 (1-II)
235.5
120.4
249.3
65.0
245.1
Al2Cu2 (1-III)
Al2Cu2 clusters having ground state with Ms= 3
242.0
68.4
241.9
101.2
101.1
241.9
68.5
241.9
Al2Cu2 (3-I)
252.1
62.4
233.3
62.4
252.1
Al2Cu2 (3-II)
62.5
2.444
2.437
2.377
61.1
Al2Cu2 (3-III)
245.8
232.6
63.8
237.4
152.2
Al2Cu2 (3-IV)

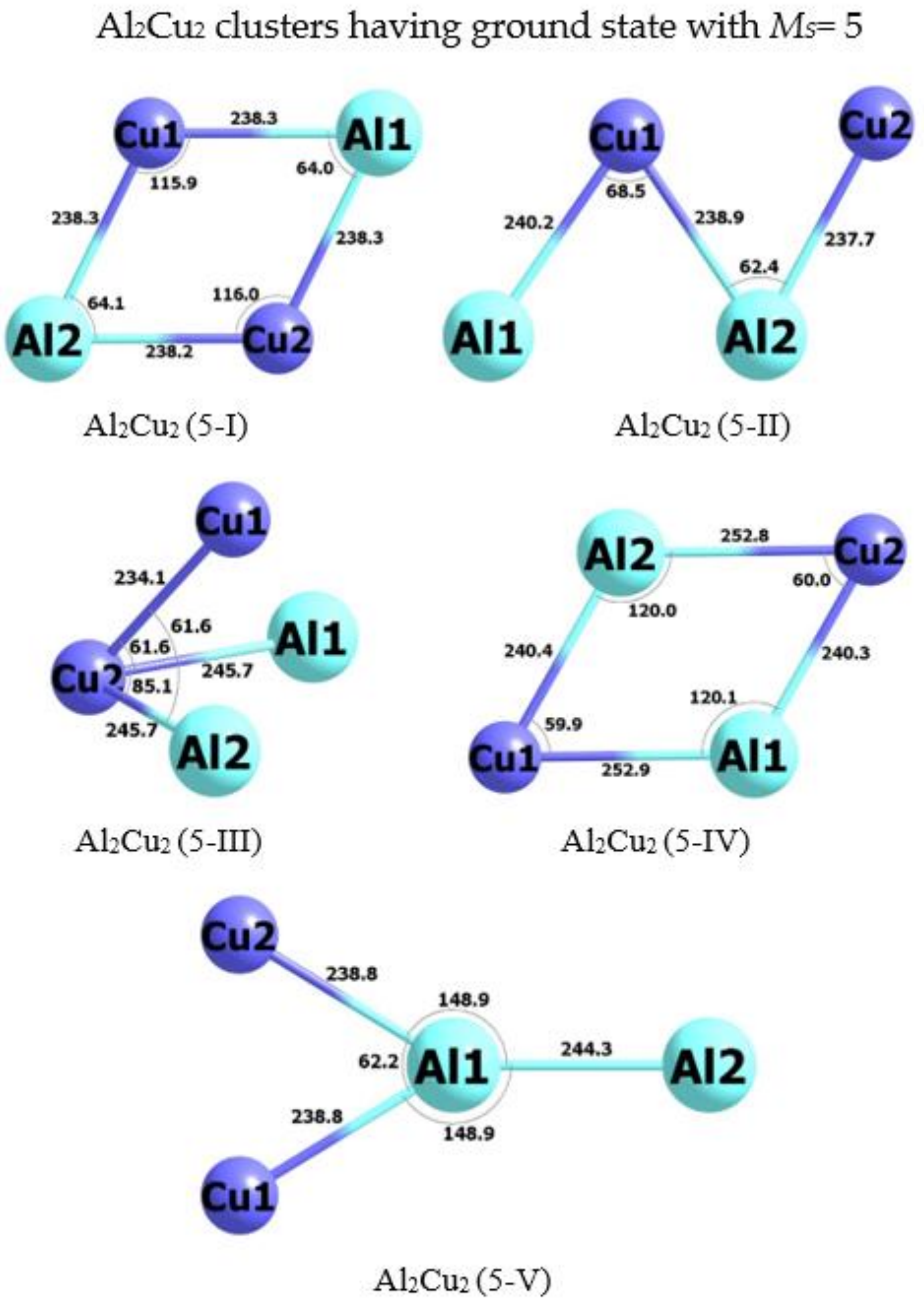

Figure S9. Molecular structures of Al_2Cu_2 metal clusters.

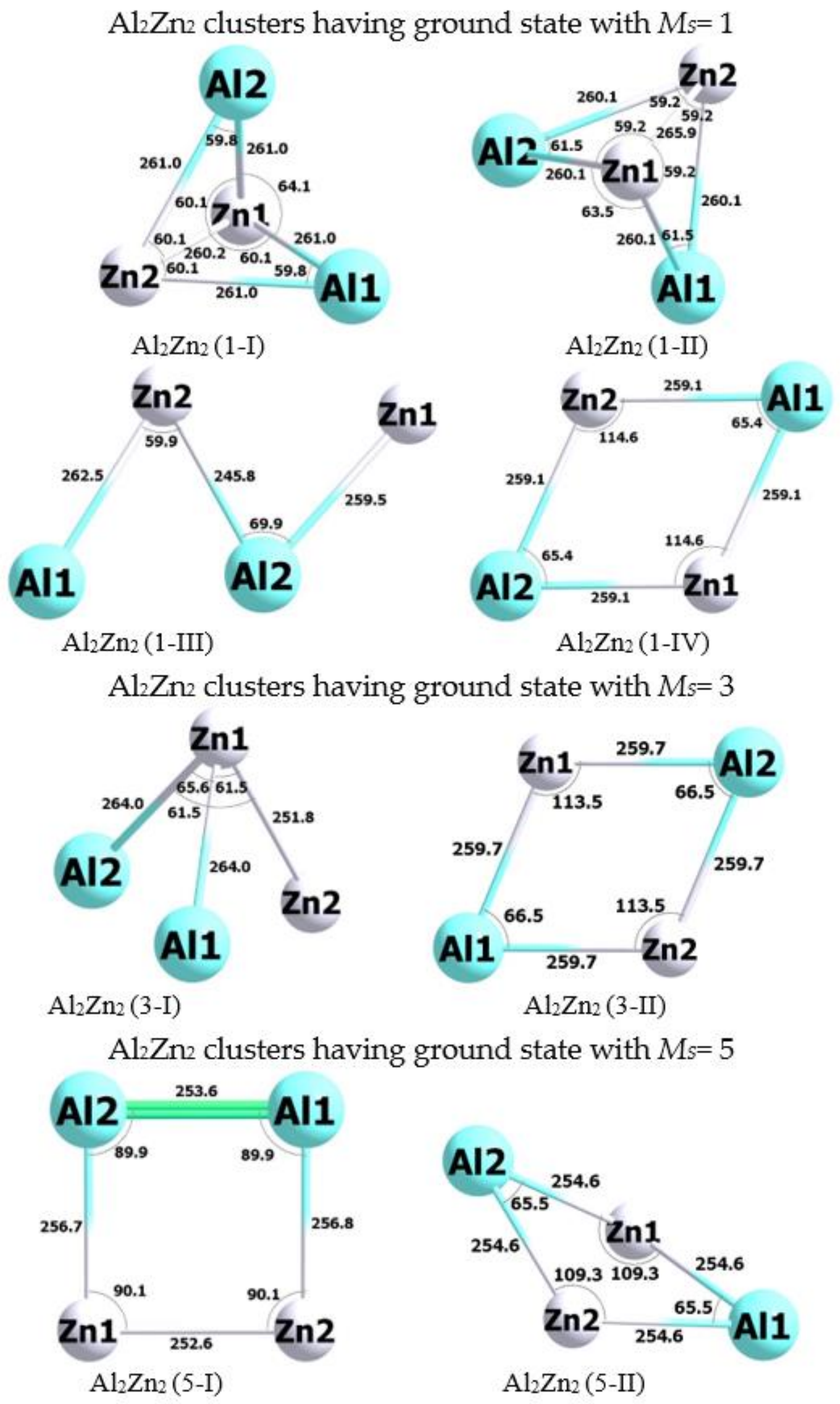

Figure S10. Molecular structures of Al_2Zn_2 metal clusters.

Table S1. Relative energies and spin multiplicities of the ground states of various isomers of Al_2Sc_2 metal clusters.

Structure Designation	Relative Energy, kJ/mol	Structure Designation	Relative Energy, kJ/mol
Metal clusters with M_S= 1		Metal clusters with M_S= 3	
Al_2Sc_2 (1-I)*	15.0	**Al_2Sc_2 (3-I)**	**0.0**
Al_2Sc_2 (1-II)*	18.7	Metal clusters with M_S= 5	
Al_2Sc_2 (1-III)	46.5	Al2Sc2 (5-I)	18.6
Al_2Sc_2 (1-IV)	51.9	Al2Sc2 (5-II)	59.4

Table S2. Relative energies and spin multiplicities of the ground states of various isomers of Al_2Ti_2 metal clusters.

Structure Designation	Relative Energy, kJ/mol	Structure Designation	Relative Energy, kJ/mol
Metal clusters with M_S= 1		Al_2Ti_2 (1-VIII)	105.0
Al_2Ti_2 (1-I)*	17.3	Metal clusters with M_S= 3	
Al_2Ti_2 (1-II)*	39.5	**Al_2Ti_2 (3-I)**	**0.0**
Al_2Ti_2 (1-III)	40.1	Al_2Ti_2 (3-II)	41.6
Al_2Ti_2 (1-IV)*	46.0	Metal clusters with M_S= 5	
Al_2Ti_2 (1-V)*	50.0	Al_2Ti_2 (5-I)	5.7
Al_2Ti_2 (1-VI)	55.2	Al_2Ti_2 (5-II)	12.7
Al_2Ti_2 (1-VII)*	90.9	Al_2Ti_2 (5-III)	14.4

Table S3. Relative energies and spin multiplicities of the ground states of various isomers of Al_2V_2 metal clusters.

Structure Designation	Relative Energy, kJ/mol	Structure Designation	Relative Energy, kJ/mol
Metal clusters with M_S= 1		Metal clusters with M_S= 3	
Al_2V_2 (1-I)*	2.7	**Al_2V_2 (3-I)**	**0.0**
Al_2V_2 (1-II)*	9.7	Al_2V_2 (3-II)	40.2
Al_2V_2 (1-III)*	13.9	Al_2V_2 (3-III)	112.5
Al_2V_2 (1-IV)*	17.0	Metal clusters with M_S= 5	
Al_2V_2 (1-V)	27.9	Al_2V_2 (5-I)	41.0
Al_2V_2 (1-VI)	33.9	Al_2V_2 (5-II)	61.5
Al_2V_2 (1-VII)*	38.1	Al_2V_2 (5-III)	100.8
Al_2V_2 (1-VIII)	42.6		

Table S4. Relative energies and spin multiplicities of the ground states of various isomers of Al_2Cr_2 metal clusters.

Structure Designation	Relative Energy, kJ/mol	Structure Designation	Relative Energy, kJ/mol
Metal clusters with M_S= 1		Al_2Cr_2 (3-II)	63.7
**Al_2Cr_2 (1-I)***	**0.0**	Al_2Cr_2 (3-III)	87.6
Al_2Cr_2 (1-II)	117.2	Metal clusters with M_S= 5	
Al_2Cr_2 (1-III)	213.7	Al_2Cr_2 (5-I)	172.1
Metal clusters with M_S= 3		Al_2Cr_2 (5-II)	180.1
Al_2Cr_2 (3-I)	32.7	Al_2Cr_2 (5-III)	192.6

Table S5. Relative energies and spin multiplicities of the ground states of various isomers of Al_2Mn_2 metal clusters.

Structure Designation	Relative Energy, kJ/mol	Structure Designation	Relative Energy, kJ/mol
Metal clusters with M_S= 1		Al_2Mn_2 (3-III)	163.1
**Al_2Mn_2 (1-I)***	**0.0**	Metal clusters with M_S= 5	
Al_2Mn_2 (1-II)*	152.5	Al_2Mn_2 (5-I)	140.6
Al_2Mn_2 (1-III)*	190.8	Al_2Mn_2 (5-II)	145.0
Al_2Mn_2 (1-IV)*	197.7	Al_2Mn_2 (5-III)	145.4
Al_2Mn_2 (1-V)	216.5	Al_2Mn_2 (5-IV)	151.8
Al_2Mn_2 (1-VI)*	250.3	Al_2Mn_2 (5-V)	161.9
Al_2Mn_2 (1-VII)*	307.4	Al_2Mn_2 (5-VI)	207.0
Al_2Mn_2 (1-VIII)	315.2	Al_2Mn_2 (5-VII)	214.3
Metal clusters with M_S= 3		Metal clusters with M_S= 7	
Al_2Mn_2 (3-I)	29.1	Al_2Mn_2 (7-I)	52.7
Al_2Mn_2 (3-II)	163.0	Al_2Mn_2 (7-II)	192.5

Table S6. Relative energies and spin multiplicities of the ground states of various isomers of Al_2Fe_2 metal clusters.

Structure Designation	Relative Energy, kJ/mol	Structure Designation	Relative Energy, kJ/mol
Metal clusters with M_S= 1		Al_2Fe_2 (3-II)	69.2
Al_2Fe_2 (1-I)*	48.7	Al_2Fe_2 (3-III)	143.8
Al_2Fe_2 (1-II)*	49.8	Al_2Fe_2 (3-IV)	147.3
Al_2Fe_2 (1-III)*	136.3	Al_2Fe_2 (3-V)	198.5
Al_2Fe_2 (1-IV)*	142.4	Al_2Fe_2 (3-VI)	236.3
Al_2Fe_2 (1-V)*	155.0	Metal clusters with M_S= 5	
Al_2Fe_2 (1-VI)	196.6	**Al_2Fe_2 (5-I)**	**0.0**
Al_2Fe_2 (1-VII)*	236.3	Al_2Fe_2 (5-II)	9.9
Al_2Fe_2 (1-VIII)*	245.5	Al_2Fe_2 (5-III)	53.5
Al_2Fe_2 (1-IX)	261.7	Al_2Fe_2 (5-IV)	55.2
Al_2Fe_2 (1-X)	307.0	Al_2Fe_2 (5-V)	70.6
Metal clusters with M_S= 3		Al_2Fe_2 (5-VI)	254.8
Al_2Fe_2 (3-I)	61.3		

Table S7. Relative energies and spin multiplicities of the ground states of various isomers of Al_2Co_2 metal clusters.

Structure Designation	Relative Energy, kJ/mol	Structure Designation	Relative Energy, kJ/mol
Metal clusters with M_S= 1		Al_2Co_2 (1-XIII)*	173.1
Al_2Co_2 (1-I)*	72.7	Al_2Co_2 (1-XIV)	241.2
Al_2Co_2 (1-II)*	75.4	Metal clusters with M_S= 3	
Al_2Co_2 (1-III)*	81.4	Al_2Co_2 (3-I)	26.3
Al_2Co_2 (1-IV)*	85.8	Al_2Co_2 (3-II)	103.8
Al_2Co_2 (1-V)*	135.2	Metal clusters with M_S= 5	
Al_2Co_2 (1-VI)	139.2	**Al_2Co_2 (5-I)**	**0.0**
Al_2Co_2 (1-VII)*	141.0	Metal clusters with M_S= 7	
Al_2Co_2 (1-VIII)	146.7	Al_2Co_2 (7-I)	103.8
Al_2Co_2 (1-IX)*	148.4	Al_2Co_2 (7-II)	116.5
Al_2Co_2 (1-X)	167.1	Al_2Co_2 (7-III)	158.7
Al_2Co_2 (1-XI)	168.4	Al_2Co_2 (7-IV)	162.0
Al_2Co_2 (1-XII)	168.5		

Table S8. Relative energies and spin multiplicities of the ground states of various isomers of Al_2Ni_2 metal clusters.

Structure Designation	Relative Energy, kJ/mol	Structure Designation	Relative Energy, kJ/mol
Metal clusters with M_S= 1		Al_2Ni_2 (3-II)	25.2
**Al_2Ni_2 (1-I)**	**0.0**	Metal clusters with M_S= 5	
Al_2Ni_2 (1-II)*	90.5	Al_2Ni_2 (5-I)	118.6
Metal clusters with M_S= 3		Al_2Ni_2 (5-II)	190.1
Al_2Ni_2 (3-I)	12.8		

Table S9. Relative energies and spin multiplicities of the ground states of various isomers of Al_2Cu_2 metal clusters.

Structure Designation	Relative Energy, kJ/mol	Structure Designation	Relative Energy, kJ/mol
Metal clusters with M_S= 1		Al_2Cu_2 (3-IV)	127.5
**Al_2Cu_2 (1-I)**	**0.0**	Metal clusters with M_S= 5	
Al_2Cu_2 (1-II)*	114.6	Al_2Cu_2 (5-I)	215.3
Al_2Cu_2 (1-III)	115.7	Al_2Cu_2 (5-II)	229.0
Metal clusters with M_S= 3		Al_2Cu_2 (5-III)	245.4
Al_2Cu_2 (3-I)	79.3	Al_2Cu_2 (5-IV)	260.7
Al_2Cu_2 (3-II)	85.8	Al_2Cu_2 (5-V)	274.6
Al_2Cu_2 (3-III)	105.7		

Table S10. Relative energies and spin multiplicities of the ground states of various isomers of Al_2Zn_2 metal clusters.

Structure Designation	Relative Energy, kJ/mol	Structure Designation	Relative Energy, kJ/mol
Metal clusters with M_S= 1		Metal clusters with M_S= 3	
		Al_2Zn_2 (3-I)	16.3
**Al_2Zn_2 (1-I)***	**0.0**	Al_2Zn_2 (3-II)	47.4
Al_2Zn_2 (1-II)	3.5	Metal clusters with M_S= 5	
Al_2Zn_2 (1-III)	45.4	Al_2Zn_2 (5-I)	82.0
Al_2Zn_2 (1-IV)*	60.9	Al_2Zn_2 (5-II)	151.3

Table S11. The values of energies (are given in eV) of highest occupied (HOMO) and lowest unoccupied (LUMO) molecular orbitals, and values of gap. The symbol "a" corresponds to electron with spin (+1/2), "b", to electron with spin (–1/2).

Cluster	HOMO (a)	LUMO (a)	Δ (a)	HOMO (b)	LUMO (b)	Δ (b)
			Al_2Sc_2			
Al_2Sc_2 (1-I)*	-3.443	-2.766	*0.677*	-3.384	-2.691	*0.693*
Al_2Sc_2 (1-II)*	-3.397	-2.773	*0.624*	-3.398	-2.773	*0.625*
Al_2Sc_2 (1-III)	-3.173	-3.098	*0.075*	-3.173	-3.098	*0.075*
Al_2Sc_2 (1-IV)	-3.159	-3.191	*-0.032*	-3.159	-3.191	*-0.032*
**Al_2Sc_2 (3-I)**	**-3.479**	**-2.842**	***0.637***	**-3.534**	**-2.617**	***0.917***
Al_2Sc_2 (5-1)	-3.321	-2.669	*0.652*	-3.839	-3.036	*0.802*
Al_2Sc_2 (5-2)	-3.303	-2.678	*0.625*	-3.386	-3.177	*0.209*
			Al_2Ti_2			
Al_2Ti_2 (1-I)*	-4.110	-3.369	*0.742*	-4.171	-3.248	*0.923*
Al_2Ti_2 (1-II)*	-3.830	-3.347	*0.482*	-3.830	-3.347	*0.482*
Al_2Ti_2 (1-III)	-3.830	-3.342	*0.488*	-3.830	-3.342	*0.488*
Al_2Ti_2 (1-IV)*	-3.793	-3.334	*0.459*	-3.793	-3.334	*0.459*
Al_2Ti_2 (1-V)*	-3.743	-3.240	*0.503*	-3.743	-3.240	*0.503*

Cluster	HOMO (a)	LUMO (a)	Δ (a)	HOMO (b)	LUMO (b)	Δ (b)
Al_2Ti_2 (1-VI)	-3.766	-3.495	*0.271*	-3.766	-3.495	*0.271*
Al_2Ti_2 (1-VII)*	-3.855	-3.045	*0.811*	-3.983	-3.278	*0.705*
Al_2Ti_2 (1-VIII)	-3.361	-3.071	*0.290*	-3.361	-3.071	*0.290*
**Al_2Ti_2 (3-I)**	**-4.212**	**-3.395**	***0.817***	**-3.998**	**-3.080**	***0.918***
Al_2Ti_2 (3-II)	-3.486	-3.238	*0.247*	-3.499	-2.582	*0.917*
Al_2Ti_2 (5-I)	-3.819	-3.433	*0.387*	-4.035	-3.318	*0.717*
Al_2Ti_2 (5-II)	-3.648	-3.364	*0.284*	-3.897	-3.049	*0.848*
Al_2Ti_2 (5-III)	-3.654	-3.482	*0.173*	-3.914	-3.116	*0.799*
			Al_2V_2			
Al_2V_2 (1-I)*	-3.854	-3.015	*0.839*	-3.854	-3.015	*0.839*
Al_2V_2 (1-II)*	-4.074	-3.557	*0.516*	-4.074	-3.557	*0.516*
Al_2V_2 (1-III)*	-3.897	-3.444	*0.453*	-3.897	-3.444	*0.453*
Al_2V_2 (1-IV)*	-4.050	-3.722	*0.328*	-4.114	-3.687	*0.427*
Al_2V_2 (1-V)	-3.857	-3.570	*0.287*	-3.857	-3.570	*0.287*
Al_2V_2 (1-VI)	-4.201	-3.984	*0.218*	-4.201	-3.984	*0.218*
Al_2V_2 (1-VII)*	-3.915	-3.287	*0.628*	-4.140	-2.957	*1.183*
Al_2V_2 (1-VIII)	-3.858	-3.250	*0.608*	-3.858	-3.250	*0.608*
**Al_2V_2 (3-I)**	**-4.157**	**-2.992**	***1.165***	**-4.010**	**-3.524**	***0.486***
Al_2V_2 (3-II)	-3.896	-3.033	*0.864*	-4.041	-3.285	*0.756*
Al_2V_2 (3-III)	-3.997	-3.814	*0.183*	-4.100	-3.661	*0.438*
Al_2V_2 (5-I)	-3.471	-2.585	*0.886*	-4.058	-3.241	*0.817*
Al_2V_2 (5-II)	-4.043	-3.151	*0.892*	-4.074	-3.654	*0.421*
Al_2V_2 (5-III)	-3.413	-2.834	*0.579*	-4.152	-3.689	*0.463*
			Al_2Cr_2			
**Al_2Cr_2 (1-I)***	**-3.831**	**-3.143**	***0.688***	**-3.831**	**-3.143**	***0.688***
Al_2Cr_2 (1-II)	-4.336	-3.480	*0.856*	-4.336	-3.480	*0.856*
Al_2Cr_2 (1-III)	-4.107	-3.281	*0.826*	-4.107	-3.281	*0.826*
Al_2Cr_2 (3-I)	-3.459	-2.607	*0.852*	-4.622	-3.489	*1.133*
Al_2Cr_2 (3-II)	-3.817	-2.923	*0.894*	-4.453	-2.836	*1.618*
Al_2Cr_2 (3-III)	-3.614	-2.743	*0.870*	-4.379	-2.971	*1.408*
Al_2Cr_2 (5-I)	-3.593	-2.821	*0.772*	-4.316	-3.443	*0.873*
Al_2Cr_2 (5-II)	-3.409	-2.779	*0.630*	-4.747	-4.119	*0.628*
Al_2Cr_2 (5-III)	-3.576	-2.716	*0.860*	-4.536	-3.612	*0.925*
			Al_2Mn_2			
**Al_2Mn_2 (1-I)***	**-3.708**	**-2.952**	***0.756***	**-3.708**	**-2.952**	***0.756***
Al_2Mn_2 (1-II)*	-3.929	-3.506	*0.423*	-3.598	-3.101	*0.498*
Al_2Mn_2 (1-III)*	-4.342	-3.389	*0.953*	-4.342	-3.389	*0.953*
Al_2Mn_2 (1-IV)*	-4.202	-3.665	*0.537*	-4.409	-3.161	*1.248*
Al_2Mn_2 (1-V)	-4.020	-3.815	*0.205*	-4.020	-3.815	*0.205*
Al_2Mn_2 (1-VI)*	-3.587	-2.601	*0.986*	-3.991	-3.849	*0.142*
Al_2Mn_2 (1-VII)*	-3.744	-3.588	*0.156*	-3.744	-3.587	*0.157*
Al_2Mn_2 (1-VIII)	-3.494	-3.338	*0.156*	-3.494	-3.338	*0.156*
Al_2Mn_2 (3-I)	-3.558	-3.136	*0.422*	-4.495	-3.441	*1.054*
Al_2Mn_2 (3-II)	-4.522	-3.620	*0.902*	-4.250	-3.344	*0.906*
Al_2Mn_2 (3-III)	-4.522	-3.619	*0.903*	-4.251	-3.344	*0.906*
Al_2Mn_2 (5-I)	-3.448	-3.273	*0.176*	-5.023	-3.710	*1.312*
Al_2Mn_2 (5-II)	-4.332	-4.143	*0.189*	-4.193	-3.307	*0.886*

Cluster	HOMO (a)	LUMO (a)	Δ (a)	HOMO (b)	LUMO (b)	Δ (b)
Al_2Mn_2 (5-III)	-4.317	-4.147	*0.171*	-4.237	-3.323	*0.913*
Al_2Mn_2 (5-IV)	-4.550	-3.927	*0.623*	-3.729	-2.854	*0.875*
Al_2Mn_2 (5-V)	-3.517	-2.615	*0.902*	-4.893	-3.594	*1.298*
Al_2Mn_2 (5-VI)	-4.244	-3.760	*0.484*	-3.713	-2.688	*1.025*
Al_2Mn_2 (5-VII)	-4.776	-3.948	*0.828*	-3.241	-2.677	*0.564*
Al_2Mn_2 (7-I)	-4.797	-3.776	*1.021*	-3.751	-3.013	*0.738*
Al_2Mn_2 (7-II)	-4.859	-3.609	*1.250*	-3.519	-3.030	*0.490*
			Al_2Fe_2			
Al_2Fe_2 (1-I)*	-3.897	-3.292	*0.604*	-3.897	-3.292	*0.604*
Al_2Fe_2 (1-II)*	-3.891	-3.256	*0.634*	-3.891	-3.256	*0.634*
Al_2Fe_2 (1-III)*	-3.488	-3.210	*0.277*	-3.940	-3.373	*0.567*
Al_2Fe_2 (1-IV)*	-4.532	-3.741	*0.792*	-4.532	-3.741	*0.792*
Al_2Fe_2 (1-V)*	-4.095	-2.981	*1.114*	-4.249	-3.629	*0.620*
Al_2Fe_2 (1-VI)	-4.011	-4.086	*-0.075*	-4.011	-4.086	*-0.075*
Al_2Fe_2 (1-VII)*	-4.073	-3.369	*0.704*	-4.074	-3.370	*0.703*
Al_2Fe_2 (1-VIII)*	-3.969	-3.408	*0.561*	-3.969	-3.408	*0.561*
Al_2Fe_2 (1-IX)	-3.631	-4.357	*-0.727*	-3.631	-4.357	*-0.727*
Al_2Fe_2 (1-X)	-3.516	-3.625	*-0.110*	-3.516	-3.625	*-0.110*
Al_2Fe_2 (3-I)	-4.651	-3.698	*0.953*	-3.761	-2.602	*1.159*
Al_2Fe_2 (3-II)	-4.385	-3.604	*0.781*	-3.972	-3.369	*0.604*
Al_2Fe_2 (3-III)	-3.946	-3.030	*0.916*	-4.067	-3.092	*0.975*
Al_2Fe_2 (3-IV)	-3.663	-3.356	*0.307*	-4.290	-3.546	*0.744*
Al_2Fe_2 (3-V)	-4.163	-3.342	*0.821*	-3.644	-3.191	*0.453*
Al_2Fe_2 (3-VI)	-4.410	-3.456	*0.954*	-3.122	-2.764	*0.358*
**Al_2Fe_2 (5-I)**	**-4.843**	**-3.723**	***1.120***	**-3.825**	**-3.555**	***0.270***
Al_2Fe_2 (5-II)	-4.737	-3.706	*1.031*	-3.720	-3.651	*0.070*
Al_2Fe_2 (5-III)	-4.198	-4.054	*0.144*	-3.961	-3.512	*0.449*
Al_2Fe_2 (5-IV)	-4.720	-3.141	*1.580*	-3.568	-2.874	*0.695*
Al_2Fe_2 (5-V)	-4.686	-3.056	*1.629*	-3.251	-2.937	*0.315*
Al_2Fe_2 (5-VI)	-3.597	-2.957	*0.640*	-4.313	-3.927	*0.386*
			Al_2Co_2			
Al_2Co_2 (1-I)*	-4.374	-3.825	*0.550*	-4.313	-3.707	*0.606*
Al_2Co_2 (1-II)*	-4.304	-3.782	*0.523*	-4.304	-3.782	*0.522*
Al_2Co_2 (1-III)*	-4.473	-3.853	*0.621*	-4.421	-3.950	*0.470*
Al_2Co_2 (1-IV)*	-4.217	-3.660	*0.557*	-3.882	-3.406	*0.476*
Al_2Co_2 (1-V)*	-3.902	-3.134	*0.768*	-3.990	-3.224	*0.767*
Al_2Co_2 (1-VI)	-3.599	-3.546	*0.053*	-3.599	-3.546	*0.053*
Al_2Co_2 (1-VII)*	-3.912	-3.327	*0.585*	-4.011	-3.212	*0.799*
Al_2Co_2 (1-VIII)	-4.207	-4.644	*-0.437*	-4.207	-4.644	*-0.437*
Al_2Co_2 (1-IX)*	-3.944	-3.155	*0.789*	-3.962	-3.444	*0.518*
Al_2Co_2 (1-X)	-4.050	-4.702	*-0.652*	-4.050	-4.702	*-0.652*
Al_2Co_2 (1-XI)	-4.159	-4.872	*-0.713*	-4.159	-4.872	*-0.713*
Al_2Co_2 (1-XII)	-4.159	-4.872	*-0.713*	-4.159	-4.872	*-0.713*
Al_2Co_2 (1-XIII)*	-3.910	-3.477	*0.432*	-3.909	-3.477	*0.432*
Al_2Co_2 (1-XIV)	-3.782	-4.208	*-0.426*	-3.782	-4.208	*-0.426*
Al_2Co_2 (3-I)	-4.586	-3.822	*0.764*	-3.872	-3.394	*0.478*
Al_2Co_2 (3-II)	-4.672	-3.079	*1.593*	-3.639	-3.151	*0.488*

Cluster	HOMO (a)	LUMO (a)	Δ (a)	HOMO (b)	LUMO (b)	Δ (b)
**Al_2Co_2 (5-I)**	**-4.544**	**-3.052**	***1.492***	**-4.691**	**-4.009**	***0.682***
Al_2Co_2 (7-I)	-3.659	-2.690	*0.969*	-4.715	-4.285	*0.430*
Al_2Co_2 (7-II)	-3.693	-2.551	*1.142*	-4.692	-4.439	*0.253*
Al_2Co_2 (7-III)	-4.039	-3.096	*0.943*	-4.796	-4.263	*0.533*
Al_2Co_2 (7-IV)	-4.041	-3.020	*1.021*	-4.734	-4.206	*0.528*
			Al_2Ni_2			
**Al_2Ni_2 (1-I)**	**-4.456**	**-3.767**	***0.689***	**-4.456**	**-3.767**	***0.689***
Al_2Ni_2 (1-II)*	-3.859	-3.244	*0.615*	-3.859	-3.244	*0.615*
Al_2Ni_2 (3-I)	-4.274	-3.045	*1.229*	-4.390	-3.819	*0.571*
Al_2Ni_2 (3-II)	-4.530	-3.097	*1.433*	-4.689	-4.438	*0.251*
Al_2Ni_2 (5-I)	-3.549	-2.426	*1.123*	-4.539	-4.236	*0.303*
Al_2Ni_2 (5-II)	-3.858	-3.013	*0.845*	-4.494	-4.259	*0.235*
			Al_2Cu_2			
**Al_2Cu_2 (1-I)**	**-4.586**	**-3.311**	***1.275***	**-4.586**	**-3.311**	***1.275***
Al_2Cu_2 (1-II)*	-4.182	-3.345	*0.838*	-4.182	-3.345	*0.838*
Al_2Cu_2 (1-III)	-4.165	-3.440	*0.725*	-4.165	-3.440	*0.725*
Al_2Cu_2 (3-I)	-3.603	-2.612	*0.992*	-5.222	-3.268	*1.954*
Al_2Cu_2 (3-II)	-3.650	-2.445	*1.205*	-5.105	-4.204	*0.901*
Al_2Cu_2 (3-III)	-4.001	-3.023	*0.978*	-4.954	-3.245	*1.709*
Al_2Cu_2 (3-IV)	-3.888	-2.720	*1.168*	-4.775	-3.449	*1.326*
Al_2Cu_2 (5-I)	-3.922	-2.831	*1.090*	-6.006	-3.816	*2.190*
Al_2Cu_2 (5-II)	-3.601	-2.558	*1.043*	-5.662	-4.192	*1.470*
Al_2Cu_2 (5-III)	-3.317	-3.000	*0.318*	-5.610	-4.080	*1.529*
Al_2Cu_2 (5-IV)	-3.673	-3.070	*0.602*	-5.751	-4.984	*0.767*
Al_2Cu_2 (5-V)	-3.377	-2.977	*0.400*	-5.930	-3.598	*2.333*
			Al_2Mn_2			
**Al_2Zn_2 (1-I)***	**-3.718**	**-2.805**	***0.913***	**-3.718**	**-2.805**	***0.913***
Al_2Zn_2 (1-II)	-3.618	-2.939	*0.680*	-3.618	-2.939	*0.680*
Al_2Zn_2 (1-III)	-3.837	-3.410	*0.427*	-3.837	-3.410	*0.427*
Al_2Zn_2 (1-IV)*	-4.020	-3.319	*0.701*	-4.020	-3.319	*0.701*
Al_2Zn_2 (3-I)	-3.403	-2.876	*0.527*	-5.824	-3.012	*2.812*
Al_2Zn_2 (3-II)	-4.045	-2.950	*1.096*	-4.926	-3.192	*1.734*
Al_2Zn_2 (5-I)	-4.164	-2.333	*1.832*	-6.359	-3.380	*2.978*
Al_2Zn_2 (5-II)	-3.667	-2.847	*0.821*	-5.593	-3.914	*1.679*
